# Primary‐Stage Colon Cancer Impairs Muscle Energy Metabolism by Suppressing Mitochondrial Complex I Activity

**DOI:** 10.1002/jcsm.70117

**Published:** 2025-11-12

**Authors:** Xiaolin Li, Miranda van der Ende, Hanneke Moonen, Rogier Plas, Susanne Lotstra, Mieke Poland, Jaap Keijer, Renger F. Witkamp, Tjarda van Heek, Sander Grefte, Klaske van Norren, the COMUNEX group, Flip M. Kruyt, Colin Sietses, Gabie M. de Jong, Roland M. H. G. Mollen, Joé L. P. Kolkert, Dik Snijdelaar, Marlieke Visser, Jaap Dronkers

**Affiliations:** ^1^ Division of Human Nutrition and Health Wageningen University Wageningen the Netherlands; ^2^ Human and Animal Physiology Wageningen University Wageningen the Netherlands; ^3^ Gelderse Vallei Hospital Ede the Netherlands

**Keywords:** colon cancer, mitochondria oxidative respiration, mitochondrial function, skeletal muscle function

## Abstract

**Background:**

Colon cancer (CC), the third most common cancer worldwide, is accompanied by cachexia in 30% of patients. Its associated muscle loss directly impairs therapeutic response and survival. Early intervention is crucial, yet the underlying mechanisms of early‐stage muscle dysfunction remain unclear. This study investigates mitochondrial function in skeletal muscle across different CC stages to identify early metabolic alterations.

**Methods:**

The present study investigated mitochondrial function in *rectus abdominus* muscle biopsies from 30 patients with primary CC (83% male, mean age 67 ± 8 years), 10 patients with colorectal cancer with liver metastases (50% male, mean age 69 ± 6 years), and 17 age‐matched controls (65% male, mean age 66 ± 7 years). Mitochondrial oxygen consumption was assessed using high‐resolution respirometry, and transcriptional profiles were analysed via RNA sequencing.

**Results:**

Patients with primary CC exhibited reduced complex I activity compared to controls (9.02 vs. 12.47 pmol/s/mg, *p* < 0.001), accompanied by transcriptional upregulation of oxidative phosphorylation (OXPHOS)‐related genes. In contrast, patients with liver metastases showed more severe mitochondrial dysfunction, with reductions in both complex I (7.38 vs. 9.65 pmol/s/mg, *p* < 0.01) and complex II (8.36 vs. 19.73 pmol/s/mg, *p* < 0.05), but without the compensatory transcriptional upregulation seen in primary CC. These mitochondrial impairments occurred before detectable declines in physical function or systemic inflammation (C‐reactive protein, albumin).

**Conclusions:**

Our findings reveal stage‐specific mitochondrial dysfunction in CC, with early complex I impairment and a transient transcriptional adaptation in primary CC. These alterations precede clinical cachexia, suggesting mitochondrial dysfunction as a potential early biomarker for cancer‐induced muscle loss and a target for early intervention.

## 1 Introduction

Cancer cachexia is a complex condition characterized by significant muscle loss [1, 2], affecting up to 80% [3] of patients with advanced‐stage cancer, particularly those with gastrointestinal (GI) and lung cancers [4, 5]. Among cancers of the GI tract, colon cancer (CC) is the most prevalent [5], with approximately 30% of patients exhibiting cachexia at diagnosis [4, 5]. This syndrome compromises quality of life and treatment outcomes, with tumour resection often exacerbating muscle depletion and impairing postoperative recovery [5]. Early intervention is crucial for effective cachexia management, highlighting the need for early diagnostic markers and a better understanding of early‐stage disease progression. In the Netherlands, colorectal cancer screening enables early diagnosis before cachexia becomes clinically apparent. This provides an opportunity to compare the pathophysiological events in muscle tissue between early‐stage CC (from patients diagnosed through a population screening program) and later‐stage colorectal cancer (patients with liver metastases).

Impaired muscle function is not yet included in the definition and diagnosis of cachexia. This contrasts with the definition of age‐related sarcopenia, where loss of function is considered as important as loss of muscle mass [6]. This inconsistency partly stems from the lack of reliable endpoint measures of muscle function. A recent systematic review of clinical studies [7] on physical function in cancer cachexia shows that there is still no consensus on the best diagnostic measurement of physical function, although handgrip strength is mostly used. One of the underlying mechanisms of muscle function loss during cancer cachexia is assumed to be mitochondrial dysfunction [3]. An assumption primarily based on investigations in animal cancer cachexia studies [5], showing decreased mitochondrial oxygen consumption and impaired oxidative phosphorylation (OXPHOS), reduced activity of OXPHOS complex IV [8], and complex II [9], diminished ATP synthesis [10] and mitochondrial uncoupling [10]. However, human studies measuring mitochondrial function in skeletal muscle during primary‐stage cancer remain scarce. The available human studies particularly focus on patients experiencing weight loss, which limits our understanding of mitochondrial dysfunction in early disease states. An important challenge here is finding suitable controls to perform molecular and functional analyses.

In animal studies [5, 11], non‐diseased (sham) control animals can be used. This raises the question of how it could best be done in the case of patients. One option could be the inclusion of weight‐stable patients with cancer as controls [12]. Alternatively, comparisons within individuals at different time points [13] or correlating gene expression with the extent of weight loss [14] are commonly used approaches. However, a potential issue when using weight‐stable patients with cancer is that cachexia starts as a subtle and slowly developing process, which means mitochondrial dysfunction and other metabolic alterations may already be latently present in these patients. Therefore, we chose for our study to include an age‐matched control group of surgical patients who, based on their medical indication, would logically not exhibit systemic inflammation and cachexia, such as patients undergoing inguinal hernia repair, cholecystectomy or abdominal hysterectomy.

The primary aim of this observational study was to explore alterations in muscle mitochondrial function in patients with primary CC compared to age‐matched non‐cancer control patients. Additionally, we included patients with colorectal cancer with liver metastases to investigate differences in outcome parameters across various stages of CC. Our goal was to examine the interrelationship of these parameters, with the ultimate objective of identifying mitochondrial dysfunction at a pathophysiological level. This could facilitate the early detection of cancer‐induced muscle dysfunction, muscle mass loss, chronic fatigue and cachexia.

## 2 Methods

### 2.1 Compliance With Ethical Standards

The study was approved by the Medical Ethics committee from Wageningen University & Research and conducted in accordance with the principles of the Declaration of Helsinki (Fortaleza, Brazil 2013) and according to national law (WMO, The Hague, 1998). The study is registered at clinicaltrials.gov (NCT03789136). All participants were informed verbally and in writing about all experimental procedures, including possible risks and discomforts. All participants provided written informed consent before testing.

### 2.2 Study Population

This study included three groups: 17 control patients (11 males: seven with inguinal hernia and four undergoing cholecystectomy; six females: one with inguinal hernia, four undergoing cholecystectomy and one undergoing abdominal hysterectomy), 30 patients with primary CC (25 males and five females) and 10 patients with colorectal cancer with liver metastases (five males: four originating from CC and one from rectal cancer; five females: four originating from CC and one from rectal cancer). Of the patient with liver metastases, one patient was diagnosed synchronic with both the primary tumour and the metastases. The other patients were diagnosed with liver metastases in a follow‐up screening after the initial diagnosis of CRC. Exclusion criteria included recent chemotherapy or abdominal surgery within the past 6 months and malabsorption for patients with cancer, while age‐matched controls were excluded if they had prior or current tumour treatment.

Details of study population are shown in Supplementary File 1.

### 2.3 Physical Data and Biochemical Blood Markers Collection

Details of physical data and biochemical blood markers collection are shown in Supplementary File 1.

### 2.4 Muscle Transcriptome Analysis

Details of sample collection and RNA sequencing are shown in Supplementary File 1.

### 2.5 Muscle Mitochondria Oxygen Consumption

#### 2.5.1 Collecting Muscle Biopsy

Muscle tissues collected for the mitochondrial oxygen consumption test included four control patients (male patients: *N* = 2, female patients: *N* = 2), seven patients with primary CC (male patients: *N* = 4, female patients: *N* = 3) and five patients with liver metastases (male patients: *N* = 2, female patients: *N* = 3). During the surgery, a biopsy (1 cm^3^) of the *rectus abdominus* muscle was taken. Biopsies were taken directly after the first incision of the surgery to minimize the possible effect of anaesthetics. Most of the primary colon tumour resections were done using laparoscopic surgery. In the case of open surgery (most of the liver resections and surgery of controls), we took the muscle biopsy at once. Muscle biopsies for mitochondrial oxygen consumption were stored according to the procedures as described in the next paragraph. The remaining muscle material was stored at Wageningen University according to the ATMP legislation.

#### 2.5.2 Oroboros High‐Resolution Oxygraph‐2k Measurement

The mitochondrial oxygen consumption analysis was carried out in a subgroup of patients, including 13 patients with primary CC, seven patients with liver metastases and four control patients (two male + two female). Approximately 4–10 mg of muscle was immediately placed in ice‐cold biopsy preservation solution (BIOPS, 10 mM Ca‐EGTA buffer, 0.1 μM free calcium, 20 mM imidazole, 20 mM taurine, 50 mM K‐MES, 0.5 mM DTT, 6.56 mM MgCl_2_, 5.77 mM ATP, 15 mM phosphocreatine, pH 7.1) [14] for determination of mitochondrial oxygen consumption. The biopsy was kept on ice and within a maximum of 1 h of collection, muscle fibres were mechanically separated using pointed forceps under a binocular microscope in 2 mL ice‐cold BIOPS on ice. The permeabilization of the plasma membrane occurred in BIOPS supplemented with 50 μg/mL of saponin (Sigma‐Aldrich, St Louis, USA) for 30 min on ice. After the saponin incubation, muscle fibres were washed for 3 × 10 min in mitochondrial respiration medium (MiR05 kit; Oroboros Instruments, Austria) on ice. Next, muscle tissue was dried for 40 s on Whatman filter paper and subsequently weighed. Mitochondrial oxygen consumption was then measured in 2–5 mg of muscle tissue per chamber. Measurements were done in duplicate in MiR05 at 37°C using the high‐resolution Oxygraph‐2k (Oroboros Instruments, Austria), according to the following protocol. The chamber volume was set to 2 mL, the temperature within the chamber was set to 37°C, and the stirring speed was set to 750 rotations per minute. Oxygen concentration is continuously measured, recorded and used to calculate oxygen flux per milligram of muscle tissue using DatLab Software 4.3 (Oroboros Instruments, Austria). Oxygen concentration in the chamber was maintained between 275 and 450 mM to avoid potential oxygen diffusion limitation. The order and amount of substrates added to the chamber (final concentrations) are shown in Table 1. Pyruvate and malate were used to obtain complex I‐linked leak (LEAK‐N) oxygen consumption. Then, after the addition of ADP + Mg^2+^, cytochrome C was added to test the integrity of the outer mitochondrial membrane. Samples showing increased oxygen flux of > 10% upon the addition of cytochrome c were excluded. Glutamate was added to induce complex I‐linked (OXPHOS‐N) oxygen consumption. Succinate was then added to determine complex I + II‐linked (OXPHOS‐NS) oxygen consumption. Titration of CCCP was used to obtain maximum uncoupled oxygen consumption (ET‐NS). Finally, rotenone was used to inhibit complex I to be able to measure complex II‐linked (ET‐S) oxygen consumption and antimycin A was used to inhibit complex III.

**TABLE 1:** Substrate injection protocol of Oroboros.

Injection order	Substrate	Concentration	Volume/chamber
1	Pyruvate	5 mM	5 μL
	Malate	2 mM	10 μL
2	ADP + Mg^2+^	5 mM	20 μL
3	Cytochrome C	10 μM	5 μL
4	Glutamate	10 mM	10 μL
5	Succinate	10 mM	20 μL
6	CCCP	0.5 mM	Titration with 1 μL/step
7	Rotenone	2.5 μM	1 μL
8	Antimycin A	5 μM	2 μL

### 2.6 Statistics

All statistical analyses were performed in GraphPad Prism 9.5.1 software. Differences between groups were assessed with one‐way or two‐way analysis of variance (ANOVA), followed by Bonferroni multiple comparison test. Correlation analyses were performed after the assessment of normality using Shapiro–Wilk tests, demonstrating that all data analysed followed a Gaussian distribution (Pearson correlation, parametric data). Data were presented as means ± standard error of the mean (SEM). For all analyses, a *p*‐value of < 0.05 was considered to be statistically significant.

### 2.7 Data, Materials and Software Availability

The datasets used and/or analysed during the current study will be available from the corresponding author upon reasonable request.

## 3 Results

### 3.1 Physical Characteristics Are Similar Between All Study Populations

The study population consisted of control patients (*N* = 17), patients with primary CC (*N* = 30) and patients with colorectal cancer with liver metastases (*N* = 10), as outlined in Table 2. Basic physical characteristics, including age, sex and body mass index (BMI), exhibited no significant differences among the groups. Notably, patients with primary CC demonstrated a modest increase in body weight during the preceding 6 months, by 0.4% (*p* ≤ 0.05), while patients with liver metastases showed a decrease of 2.8% in body weight in the preceding 6 months (*p* ≤ 0.05).

**TABLE 2:** Physical characteristics of patients.

Variable	Control patients (*N* = 17)		Patients with primary colon cancer (*N* = 30)		Patients with liver metastases (*N* = 10)	
	Median	SD	Median	SD	Median	SD
Age (years, continuous)	67.9	7.1	68.4	6.2	64.7	10.5
Sex (male %)	65%		83%		50%	
Male	*N* = 11		*N* = 25		*N* = 5	
Female	*N* = 6		*N* = 5		*N* = 5	
Body weight						
Body mass index (BMI) (kg/m^2^)	28.1	4.8	26.5	3.6	27.7	5.2
BMI categories^a^, *N* (%)						
< 20	*N* = 0	0%	*N* = 0	0%	*N* = 0	0%
20–21.9	*N* = 2	12%	*N* = 2	7%	*N* = 1	10%
22–24.9	*N* = 2	12%	*N* = 8	27%	*N* = 3	30%
25–27.9	*N* = 6	35%	*N* = 13	43%	*N* = 1	10%
≥ 28	*N* = 7	41%	*N* = 7	23%	*N* = 5	50%
Overweight or obese, N (%)	*N* = 13	76%	*N* = 20	67%	*N* = 6	60%
Weight change past 6 months (kg)			0.5	3.6	−1.7	6.9
Weight change past 6 months (%)^b^			0.4%	4.0	−2.8%	10.3
FAACT functional assessment						
Trial outcome index (TOI)	84.8	11.7	89.7	8.9	84.7	9.4
Physical well‐being (PWB)	26.3	2.5	26.7	1.7	26.0	1.4
Functional well‐being (FWB)	18.9	6.9	21.5	5.5	18.3	7.8
Anorexia/cachexia subscale (A/CS)	39.6	5.0	41.4	3.6	40.4	3.7
Handgrip strength (kgf)	38.8	10.6	42.7	9.2	34.4	11.1
Male	43.9	7.2	43.0	9.3	44.0	5.6
Female	25.3	3.1	31.0	7.1	24.8	3.3
Maximal inspiratory pressure (MIP) (cm H_2_O)			83.9	23.3	75.0	26.9
Sit‐to‐stand (sec)			13.5	5.5	8.8	2.5
Time up and go (TUG) (sec)			6.1	1.6	4.9	
Steep ramp						
Maximum power output (Watt)	275.0	35.4	244.5	73.4	270.0	0.0
VO_2_ max (mL/kg/min)			28.8	5.6	31.3	4.4

^a^ BMI category calculated based on Martin et al. [12].

^b^ % weight loss calculated as: [(current weight − previous weight)/previous weight in kg]*100.

The handgrip strength and all elements of the FAACT questionnaire, the PWB, FWB, A/CS and the TOI (for abbreviations, see Table 2) exhibited no statistically significant differences between the groups. Maximal inspiratory pressure (MIP) was notably lower, 83.9 cm H_2_O and 75.0 cm H_2_O, in patients with primary CC and patients with liver metastases, respectively, compared to the reference values for the corresponding age group (92.7 cm H_2_O of 60–69 years for men) [15]. Additionally, the Time Up and Go (TUG) test results for all patients with CC were below the average of the reference (8.1 s, 60–69 years) [16], with an overall average of 6.1 s for patients with primary CC and 4.9 s for patients with liver metastases.

In summary, both patients with primary CC and those with liver metastases would be classified as non‐cachexic or pre‐cachexic according to the standard criteria for cancer cachexia [1, 17] and demonstrated comparable physical characteristics to control patients when assessed through physical performance tests. However, their physical performance, including the results of the MIP and TUG tests, was below age‐adjusted reference values.

### 3.2 Blood Parameters Indicate Slight Changes in C‐Reactive Protein (CRP), Albumin and Fasting Glucose During CC Progression

In patients with primary CC and patients with liver metastases, haematocrit, erythrocytes, mean corpuscular volume of erythrocytes (MCV), plasma calcium, plasma cholesterol, triglycerides and magnesium all fell within reference ranges, showing no significant differences from control patients (Table 3).

**TABLE 3:** Hematologic parameters of patients.

Variable	Reference	Control patients (*N* = 17)		Patients with primary colon cancer (*N* = 30)		Patients with liver metastases (*N* = 10)	
		Median	SD	Median	SD	Median	SD
Haemoglobin (mmol/L)		9.17	1.20	8.40	1.32	8.34	1.39
Male	8.5–11.0	9.76	0.83	8.49	1.38	9.26	1.04
Female	7.5–10.0	8.18	1.10	8.00	0.99	7.42	1.07
Haematocrit (L/L)		0.43	0.05	0.56	0.85	0.39	0.06
Male	0.41–0.51	0.46	0.04	0.60	0.94	0.43	0.05
Female	0.36–0.47	0.39	0.05	0.38	0.03	0.35	0.04
Erythrocyte (/pL)^a^		5.05	0.66	4.63	0.46	5.71	3.66
Male	4.3–6.0	5.31	0.65	4.65	0.48	4.79	0.51
Female	3.8–5.5	4.53	0.27	4.56	0.35	6.44	5.02
Mean corpuscular volume of erythrocytes (MCV) (fL)^b^	2–98	86.19	5.99	85.37	6.31	87.10	4.23
Calcium (mmol/L)	2.10–2.55	2.39	0.16	2.32	0.10	2.23	0.45
Glucose (mmol/L)	4.0–6.4	5.65	0.80	6.44	2.95	6.74	2.13
Albumin (g/L)	35–55	40.73	2.84	37.33	3.97	37.56	6.67
Cholesterol (mmol/L)	0–6.5	4.63	0.86	4.62	0.81	4.51	1.02
Triglyceride (mmol/L)	0.6–2.2	2.01	1.79	1.29	0.69	1.69	0.81
Magnesium (mmol/L)	0.7–1.0	0.83	0.16	0.83	0.08	0.82	0.06
C‐reactive protein (CRP) (mg/L)^c^	< 10	5.50	5.02	6.30	11.49	35.11	69.76
< 10		*N* = 16	94%	*N* = 28	93%	*N* = 7	70%
10–100		*N* = 1	6%	*N* = 2	7%	*N* = 2	20%
> 100		*N* = 0	0%	*N* = 0	0%	*N* = 1	10%

^a^ pL = 10–12 L.

^b^ fL = 10–15 L.

^c^ CRP subsets adjusted based on Martin et al. [19].

**FIGURE 1:**
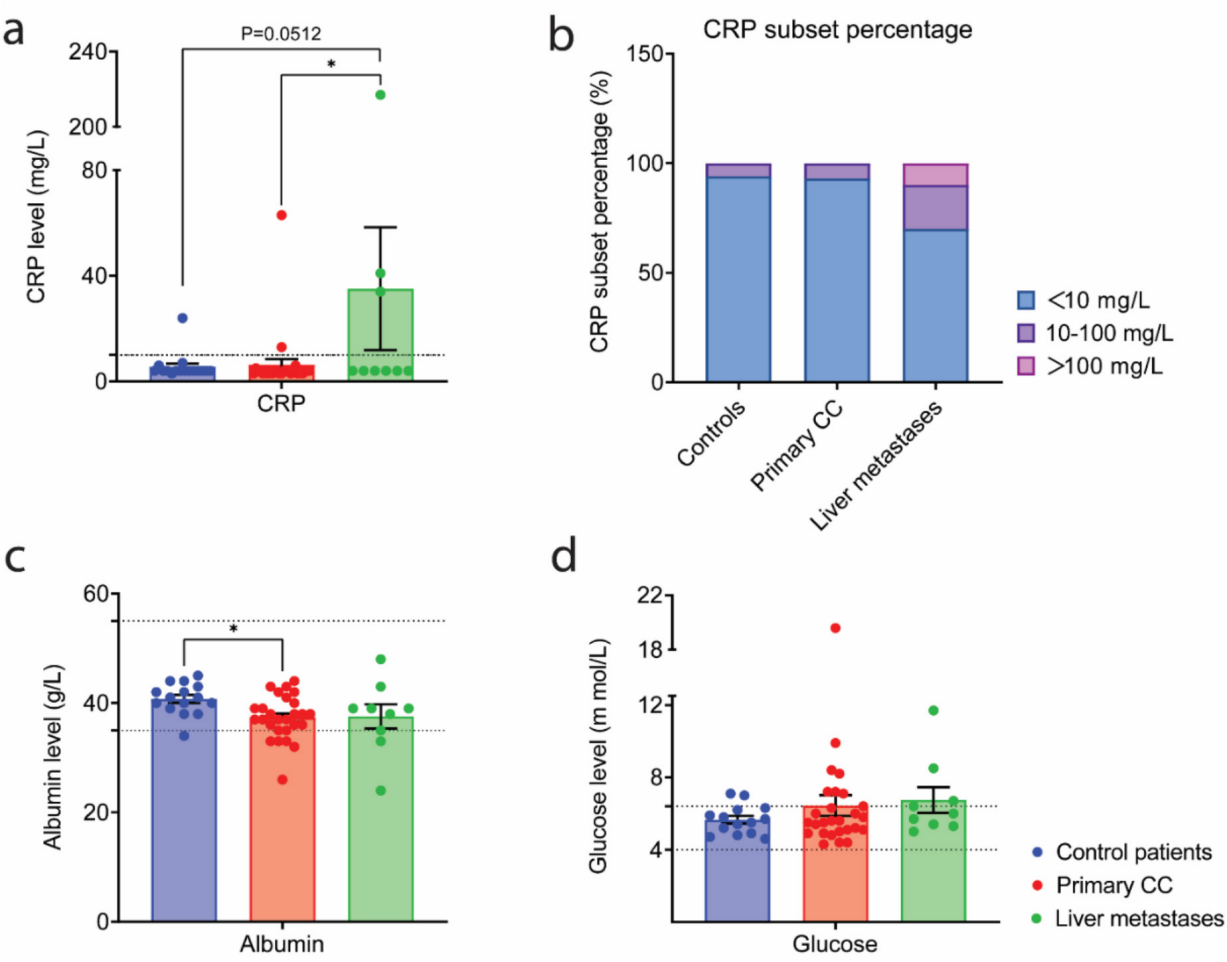
Conventional biochemical parameters in plasma. Plasma concentration of (a) C‐reactive protein (CRP) and (b) percentage of CRP subsets adjusted into < 10, 10–100, > 100 mg/L based on Martin et al. [19], (c) plasma concentrations of albumin and (d) fasting glucose measured in control patients, patients with primary colon cancer and patients with liver metastases. Control patients: *N* = 17 (male patients: *N* = 11, female patients: *N* = 6); patients with primary colon cancer: *N* = 30 (male patients: *N* = 25, female patients: *N* = 5); patients with liver metastases: *N* = 10 (male patients: *N* = 5, female patients: *N* = 5). All data were expressed as mean ± SEM Data represented in (a), (c), (d) were analysed by one‐way ANOVA with Tukey post hoc test. *p ≤ 0.05, **p ≤ 0.01 and ***p ≤ 0.001, ****p ≤ 0.0001.

As an indicator of systemic inflammation, the average CRP plasma level (Figure 1a) was 5.5 mg/L in control patients and 6.30 mg/L in patients with primary CC. However, patients with liver metastases showed an elevated CRP level of 35.11 mg/L, exceeding the reference value (< 10 mg/L) and being significantly higher than that of patients with primary CC (*p* ≤ 0.05). The adjusted subset percentage of CRP levels (Figure 1b) [17] revealed that 94% of control patients, 93% of patients with primary CC and 70% of patients with liver metastases fell within the reference range. The subset with CRP levels ranging from 10 to 100 mg/L included 6% of control patients, 7% of patients with primary CC and 20% of patients with liver metastases. Notably, one patient with liver metastases exhibited a CRP level of 217 mg/L.

Albumin (Figure 1c) is a negative acute‐phase protein utilized as an inflammatory biomarker. Albumin levels were significantly lower in patients with primary CC compared to control patients (37.33 vs. 40.73 g/L respectively, *p* ≤ 0.05). In patients with liver metastases, albumin levels were close to those of patients with primary CC (37.56 vs. 37.33 g/L respectively), but not significantly lower than in control patients. The average glucose level (Figure 1d) in control patients and patients with primary CC fell within the reference range, with six patients with primary CC exceeding the upper limit. Patients with liver metastases had an average plasma glucose level of 6.74 mmol/L, exceeding the reference range (4.0–6.4 mmol/L).

### 3.3 Transcriptional Signatures of Muscle Mitochondrial Function Exhibit Significant Differences Between Stages of CC Progression

To investigate alterations in muscle, RNA sequencing was conducted to analyse transcriptional *rectus abdominus* muscle function signatures during different stages of CC progression. MitoCarta3.0 gene set was employed to specifically analyse mitochondrial function‐related gene expression.

**FIGURE 2:**
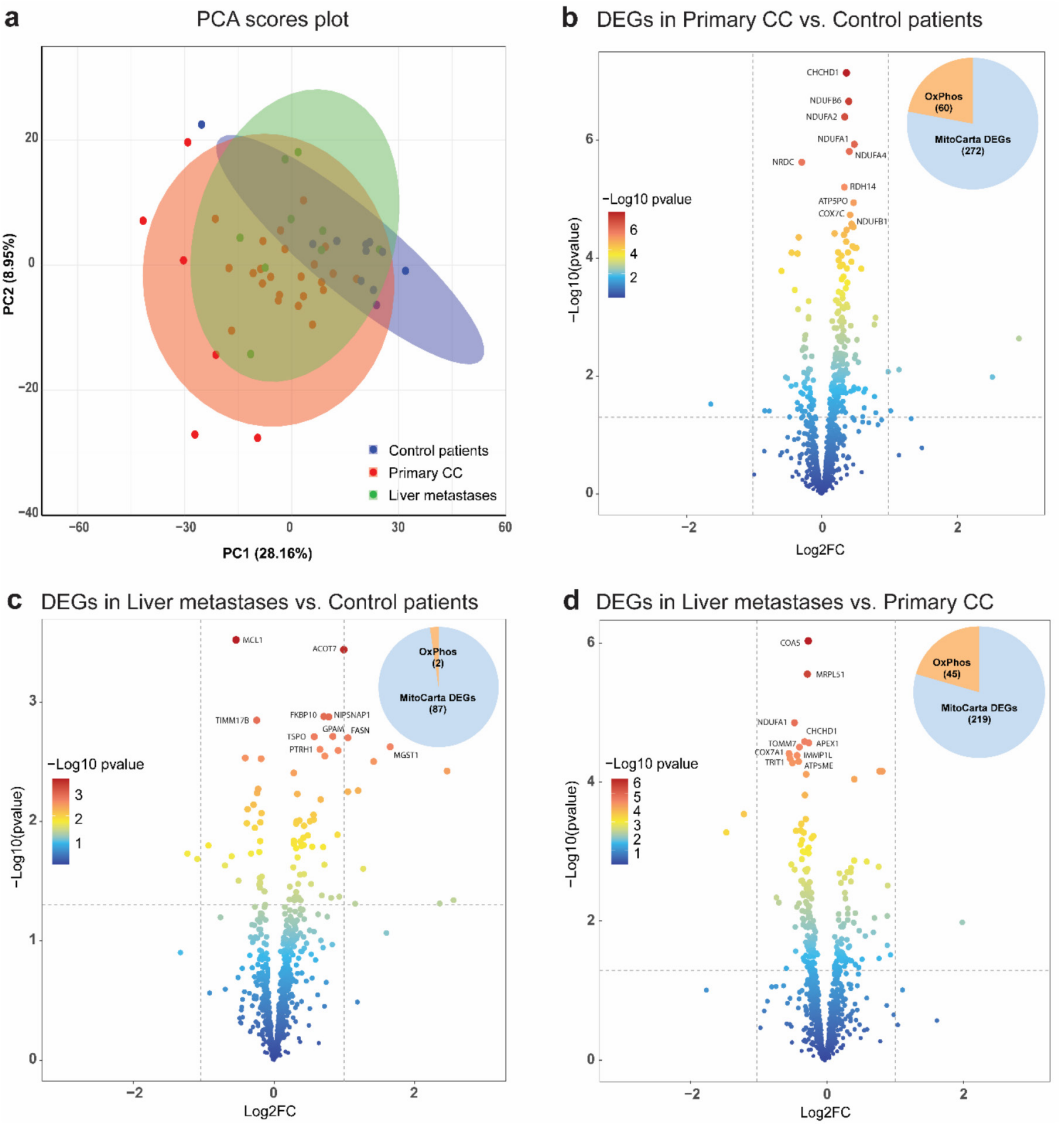
Transcriptional signatures of muscle mitochondrial function exhibit significant alterations in colon cancer progression. (a) Principal component analysis (PCA) plot of Euclidean distance between samples on variance stabilizing transformed data. Samples were coloured based on the group. Volcano plot displaying differentially expressed genes (DEGs) in MitoCarta3.0 genes set and percentage of OXPHOS‐related DEGs: (b) of primary colon cancer patients versus control patients, (c) of patients with liver metastases versus control patients, (d) of patients with liver metastases versus patients with primary colon cancer. Genes were differentially regulated using a *p*‐value of 0.05. Control patients: *N* = 11 (male patients: *N* = 7, female patients: *N* = 4); patients with primary colon cancer: *N* = 29 (male patients: *N* = 24, female patients: *N* = 5); patients with liver metastases: *N* = 9 (male patients: *N* = 5, female patients: *N* = 4).

Principal component analysis (PCA) of MitoCarta3.0 gene expression revealed distinct clustering among control patients, patients with primary CC and those with liver metastases, with principal components explaining 28.16% and 8.95% of variance, respectively (Figure 2a). Differential expression analysis (*p* < 0.05) identified 272 dysregulated genes in primary CC versus controls, with 60 (22.06%) related to OXPHOS. Among the top 10 DEGs, seven were OXPHOS‐related, including five complex I genes (*Ndufb6, Ndufa2, Ndufa1, Ndufa4, Ndufb1*) (Figure 2b). In liver metastases, 87 DEGs were detected versus controls, with two OXPHOS‐related genes (Figure 2c). Comparisons between primary CC and liver metastases identified 219 DEGs, including 45 (20.55%) linked to OXPHOS (Figure 2d).

**FIGURE 3:**
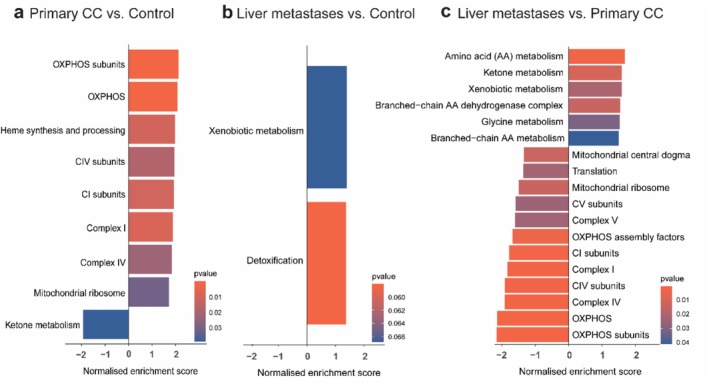
Transcriptional signatures of muscle mitochondrial function exhibit significant alterations in colon cancer progression. Gene Set Enrichment Analysis (GSEA) using mitochondrial‐related pathways: (a) of patients with primary colon cancer versus control patients, (b) of patients with liver metastases versus control patients, (c) of patients with liver metastases versus patients with primary colon cancer. Genes were compared with genes in the MitoCarta genes set using a *p*‐value of 0.05. Gene sets were filtered based on a 75% overlap in genes. Control patients: *N* = 11 (male patients: *N* = 7, female patients: *N* = 4); patients with primary colon cancer: *N* = 29 (male patients: *N* = 24, female patients: *N* = 5); patients with liver metastases: N = 9 (male patients: *N* = 5, female patients: *N* = 4).

Gene ontology analysis revealed that patients with primary CC exhibited significant (*p* < 0.05) upregulation of OXPHOS‐related pathways, including OXPHOS subunits, complexes I and IV, mitochondrial ribosome and heme synthesis, whereas ketone metabolism was downregulated (Figure 3a). Patients with liver metastases showed upregulation of xenobiotic metabolism, while detoxification pathways were downregulated (Figure 3b). Compared to primary CC, liver metastases were characterized by significant (*p* < 0.05) downregulation of mitochondrial processes, including OXPHOS, OXPHOS assembly factors, translation and mitochondrial central dogma, with concurrent upregulation of amino acid, ketone, glycine and branched‐chain amino acid metabolism (Figure 3c).

Collectively, these findings underscore distinct changes in transcriptional signatures related to muscle mitochondrial function throughout the progression of CC. Specifically, the mitochondrial oxidative respiration profile of patients with primary CC exhibited significant upregulation compared to control patients.

### 3.4 Muscle OXPHOS Transcripts Significantly Upregulated in Patients With Primary CC

To further explore changes in muscle oxidative respiration across different stages of CC, we analysed DEGs associated with key components of mitochondrial respiratory complexes, namely complexes I, II, III, IV, Cytochrome C, Coenzyme Q metabolism and the F1Fo ATP synthase (complex V).

**FIGURE 4:**
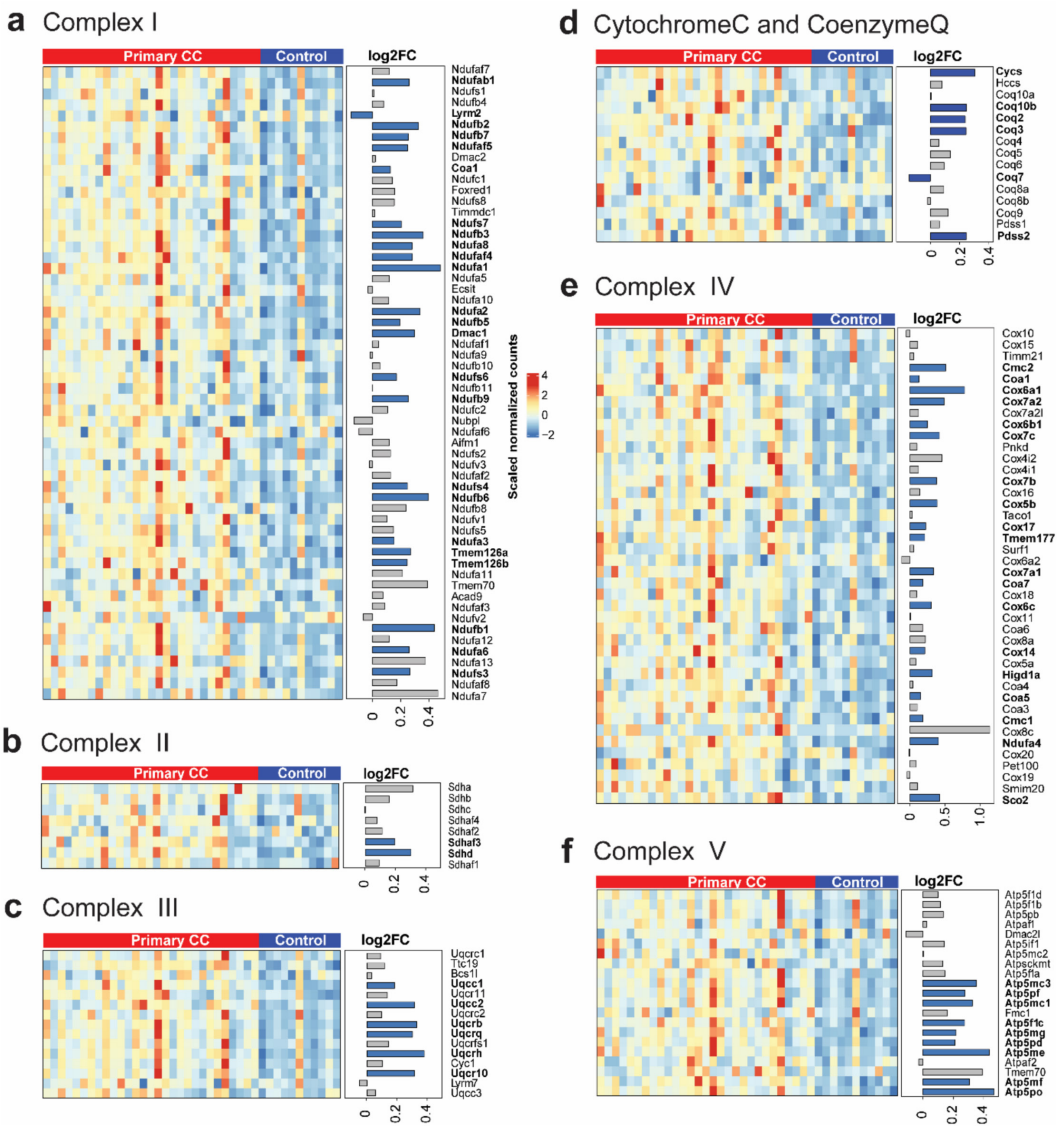
Muscle OXPHOS transcripts significantly upregulated in patients with primary CC. Heatmap displaying differentially expressed genes of (a) Complex I, (b) Complex II, (c) Complex III, (d) Cytochrome C (Cycs, Hccs) and Coenzyme Q metabolism, (e) Complex IV, (f) Complex V between patients with primary colon cancer and control patients. Genes were differentially regulated using a *p*‐value of 0.05. Control patients: *N* = 11 (male patients: *N* = 7, female patients: *N* = 4); patients with primary colon cancer: *N* = 29 (male patients: *N* = 24, female patients: *N* = 5). Colours reflect the level of gene expression.

Patients with primary CC exhibited a significant upregulation of complex I genes compared to controls (Figure 4a). Of 58 genes, 51 were upregulated, including 23 significantly (*p* < 0.05), while only one of seven downregulated genes reached significance. Notably, key ubiquinone oxidoreductase subunits (e.g., *Ndufa1–Ndufs7*) and assembly factors (*Coa1, Dmac1*) were upregulated, whereas *Lym2* was downregulated. Mitochondrial membrane proteins *Tmem126a* and *Tmem126b* also showed significant upregulation.

For complex II, *Sdhaf3* and *Sdhd* exhibited upregulation (*p* < 0.05), which are responsible for succinate‐to‐ubiquinone electron transfer (Figure 4b). In complex III, 14 out of 15 genes exhibited upregulation, with six showing significant upregulation (*p* < 0.05). The significantly upregulated genes are *Uqcc1*, *Uqcc2*, *Uqcrb*, *Uqcrq*, *Uqcrh* and *Uqcr10*, all of which are protein‐coding genes involved in the assembly of complex III (Figure 4c). Cytochrome C (*Cycs*, *Hccs*) and Coenzyme Q metabolism genes showed widespread upregulation (Figure 4d), with *Coq10b*, *Coq2*, *Coq3* and *Pdss2* significantly upregulated (*p* < 0.05), while Coq7 was downregulated (Figure 4d). These genes play critical roles in the biosynthesis and function of Coenzyme Q and Cytochrome C, both essential components of the electron transport chain in OXPHOS. Their altered expression suggests potential changes in mitochondrial function and energy production in patients with primary CC.

Complex IV genes displayed widespread upregulation, with 19 genes exhibiting significant upregulation (*p* < 0.05) out of 42 genes. Notably, cytochrome c oxidase assembly factors (Coa1, Coa5, Coa7) and genes involved in copper ion transport (Cox14, Cox17, Cox5b, etc.) were upregulated (Figure 4e). For complex V, 19 of 21 genes were upregulated, with nine genes showing significant upregulation (*p* < 0.05). Notably, this significant upregulation predominantly occurred in genes coding for ATP synthase subunits (Figure 4f).

In contrast, OXPHOS‐related DEGs in patients with liver metastases displayed minimal differences compared to control patients (Supplementary Figure S1), but exhibited a significant decrease compared to patients with primary CC (Supplementary Figure S2). To validate the RNA sequencing findings, we performed qPCR analysis of representative OXPHOS genes, including *Ndufa1*, *Ndufb1*, *Sdhd*, *Uqcrh*, *Cox5b* and *Atp5me* (Supplementary Figure S3). qPCR expression levels were strongly correlated with RNA sequencing results (r = 0.4952, *p* < 0.0001). These findings indicate a transcriptional upregulation of muscle oxidative respiration in patients with primary CC, particularly evident in mitochondrial respiratory complex I. However, the transcriptional profile of muscle oxidative respiration in patients with liver metastases showed minimal alterations compared to control patients.

### 3.5 Mitochondrial Function Is Impaired in Both Patients With Primary CC and Patients With Liver Metastases

To further assess the transcriptional findings, mitochondrial oxygen consumption in *rectus abdominus* muscle fibres was examined, providing further insight into alterations in muscle mitochondrial function across the patient groups. Representative traces of real‐time oxygen consumption of *rectus abdominus* muscle in response to specific substrates and inhibitors are shown in Supplementary Figure S3.

**FIGURE 5:**
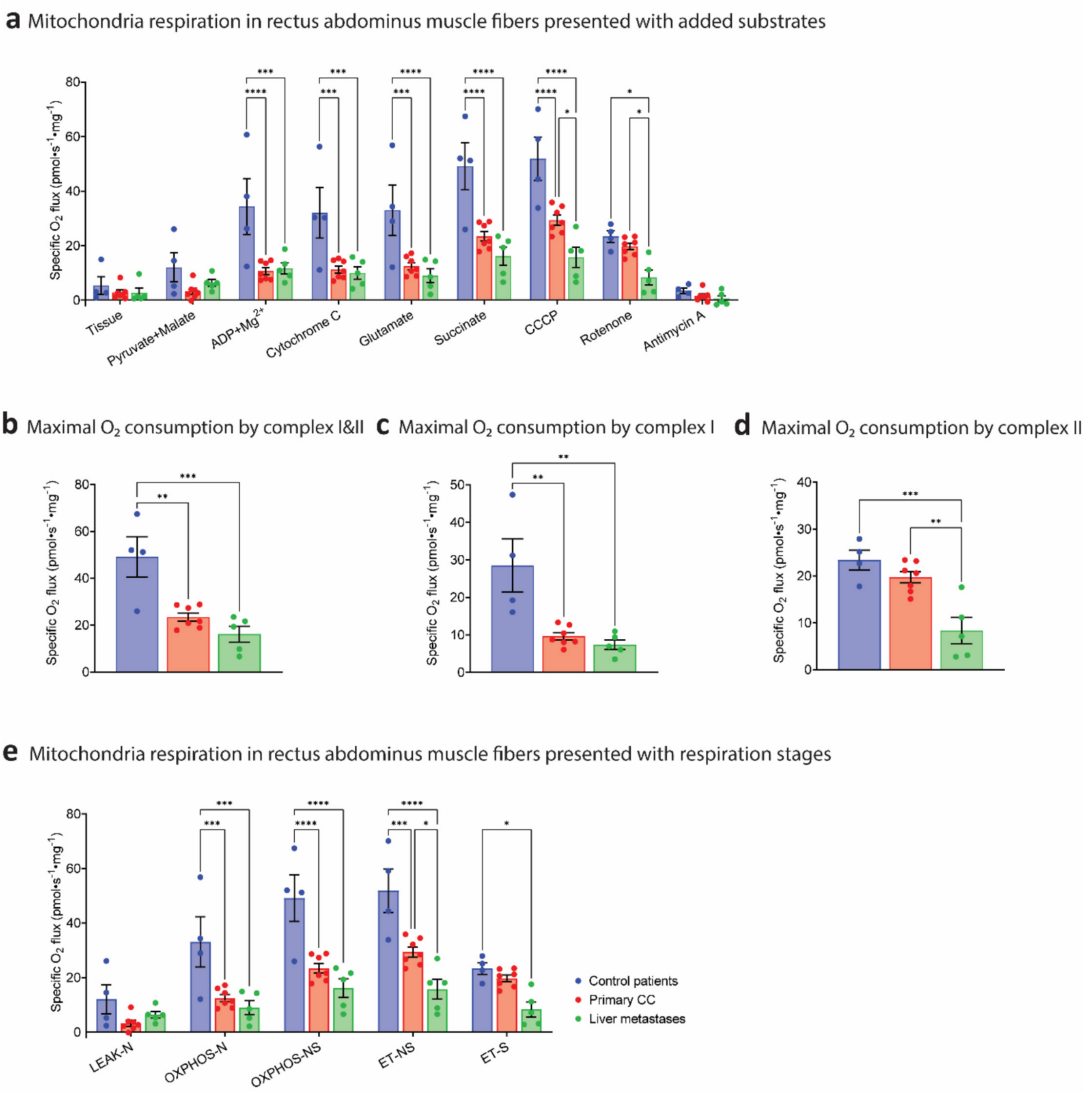
High‐resolution mitochondrial oxygen consumption in *rectus abdominus* muscle fibres of different patients. (a) Mitochondrial oxygen consumption presented with added substrates: tissue, Pyruvate+Malate, ADP + Mg^2+^, Cytochrome C, Glutamate, Succinate, Carbonyl cyanide m‐chlorophenylhydrazone (CCCP), Rotenone, Antimycin A. (b) Maximal O_2_ consumption by complexes I and II, presented by absolute values of adding Succinate. (c) Maximal O_2_ consumption by complex I, calculated by delta of CCCP and Rotenone. (d) Maximal O_2_ consumption by complex II, presented by absolute values of adding Rotenone. (e) Mitochondrial oxygen consumption presented with respiration stages: LEAK‐N, OXPHOS‐N, OXPHOS‐NS, ET‐NS, and ET‐S. Muscle tissues of control patients: *N* = 4 (male patients: *N* = 2, female patients: *N* = 2); patients with primary colon cancer: *N* = 7 (male patients: *N* = 4, female patients: *N* = 3); patients with liver metastases: *N* = 5 (male patients: *N* = 2, female patients: *N* = 3). All data were expressed as mean ± SEM. Data were analysed by two‐way ANOVA with Tukey post hoc test. *p ≤ 0.05, **p ≤ 0.01 and ***p ≤ 0.001, ****p ≤ 0.0001.

Quantification of mitochondrial function showed that in the presence of the substrates pyruvate, malate, ADP and glutamate (Figure 5a: complex I substrates; Figure 5e: OXPHOS‐N), the oxygen consumption was significantly lower in patients with primary CC and in those with liver metastases when compared to control patients (12.47 vs. 9.02 pmol/s/mg vs. 33.06 pmol/s/mg, *p* < 0.001 and *p* < 0.0001). Oxygen consumption remained significantly lower in both groups of patients with CC after the addition of succinate (Figure 5a: complex I + II substrates; Figure 5e: OXPHOS‐NS). The uncoupler CCCP was added to measure maximal uncoupled oxygen consumption (Figure 5a,b) reflecting the ET‐NS stage (Figure 5e). Compared to the control patients, oxygen consumption in both patients with primary CC and patients with liver metastases was significantly lower (51.86 vs. 29.38 pmol/s/mg, *p* < 0.001 and 15.73 pmol/s/mg, *p* < 0.0001). Moreover, oxygen consumption in patients with liver metastases was significantly lower compared to those with primary CC (*p* < 0.05).

To investigate more specifically the maximal activity of complex I and complex II in the presence of the uncoupler CCCP, rotenone was added to inhibit complex I. The maximal oxygen consumption after the addition of rotenone reflected complex II activity, while the difference in maximal oxygen consumption before and after the addition of rotenone reflected complex I activity. Maximal oxygen consumption by complex I (Figure 5c) was significantly lower in patients with primary CC (9.65 pmol/s/mg, *p* < 0.01) and patients with liver metastases (7.38 pmol/s/mg, *p* < 0.01) compared to control patients (28.48 pmol/s/mg). Maximal oxygen consumption by complex II (Figure 5d) was not changed in patients with primary CC (19.73 pmol/s/mg) but was significantly lower in patients with liver metastasis (8.36 pmol/s/mg, *p* < 0.05) compared to control patients (23.38 pmol/s/mg). These results demonstrate that mitochondrial function in the *rectus abdominus* was impaired in both patients with primary CC and patients with liver metastases. More specifically, this reduction in patients with primary CC was mainly due to a decline of complex I activity, whereas in patients with liver metastases it was because of the decline of both complex I and complex II activity.

### 3.6 Correlations Between Mitochondrial Function and Physical Indices and Blood Parameters: The Potential of Mitochondrial Dysfunction in Predicting Primary Stage CC

Pearson correlation coefficients were calculated to investigate associations between all measured parameters (Supplementary Figure S5). The steep ramp maximum power output correlated with age and handgrip strength. Moreover, FAACT correlated with other physical parameters. For example, PWB showed a significant correlation with TUG (r = 0.62, *p* < 0.01, *N* = 22), and FWB correlated with handgrip strength (r = 0.54, *p* < 0.001, *N* = 35). This shows that the changes in all physical parameters are consistent with each other.

Correlations between mitochondrial function, as indicated in Figure 5e, and other study parameters with a coefficient above 0.5 are shown in Table 4. The OXPHOS‐N stage, representing complex I‐driven oxygen consumption coupled to ATP production, showed significant correlations with weight loss, MIP and MCV. The OXPHOS‐NS stage, representing complexes I and II driven oxygen consumption coupled to ATP production, correlated with weight loss (kg) (r = 0.67, *p* < 0.05, *N* = 9), steep ramp maximum volume of O_2_ (r = 0.98, *p* < 0.05, *N* = 4) and MCV (r = −0.55, *p* < 0.05, *N* = 16). The ET‐NS stage, representing maximal complexes I and II driven oxygen consumption, also showed correlations with weight loss (kg) (r = 0.68, *p* < 0.05, N = 9), physical well‐being (r = 0.64, *p* < 0.05, *N* = 10) and MCV (r = −0.56, *p* < 0.05, N = 16). The ET‐S stage, representing maximal complex II‐driven oxygen consumption, showed a significant correlation with physical activity (r = −0.9979, *p* < 0.05, N = 3) and haematological index of systemic inflammation, including albumin (r = 0.53, *p* < 0.05, N = 16) and CRP (r = −0.61, *p* < 0.05, N = 16). These correlations of mitochondrial function with physical parameters (weight and physical well‐being) and inflammatory markers (CRP and albumin) indicate that mitochondrial dysfunction is associated with alterations of conventional biochemical parameters, underscoring the potential of mitochondrial dysfunction in predicting the development of cancer‐induced muscle dysfunction and chronic fatigue.

**TABLE 4:** Pearson correlation coefficient above 0.5 between mitochondrial function and other different study parameters.

Variable	LEAK‐N			OXPHOS‐N			OXPHOS‐NS			ET‐NS			ET‐S		
	r	*p*	*N*	r	*p*	*N*	r	*p*	*N*	r	*p*	*N*	r	*p*	*N*
Weight loss 1/2 year				0.8525	0.0035**	9	0.6673	0.0496*	9	0.6802	0.0438*	9	0.6463	0.0600	9
Weight loss %				0.7831	0.0126*	9	0.6207	0.0745	9	0.6506	0.0578	9	0.6327	0.0674	9
MIP	0.5547	0.4453	4	0.9591	0.0409*	4	0.5082	0.4918	4	0.7236	0.2764	4			
Sit‐to‐stand	0.7843	0.2157	4	0.7739	0.2261	4				0.8832	0.1168	4	0.8319	0.1681	4
TUG	0.9661	0.0339*	4				−0.6078	0.3922	4	0.8641	0.1359	4	0.8630	0.1370	4
Steepramp max				0.5250	0.6481	3							0.7228	0.4858	3
Steepramp VO_2_ max				0.6826	0.3174	4	0.9769	0.0231*	4						
Energy													−0.9243	0.2493	3
Activity	−0.5733	0.6113	3										−0.9979	0.0416*	3
PWB				0.5774	0.0805	10	0.5500	0.0995	10	0.6428	0.0450*	10	0.5585	0.0933	10
MCV				−0.6191	0.0106*	16	−0.5502	0.0272*	16	−0.5602	0.0240*	16			
Albumin													0.5284	0.0354*	16
CRP													−0.6076	0.0125*	16

* *p*≤0.005.

** *p*≤0.01.

*** *p*≤0.001.

**** *p*≤0.0001.

## 4 Discussion

This study adds to our understanding of the earliest effects of tumour development on skeletal muscle tissue. To the best of our knowledge, this is the first study in patients with cancer demonstrating that signals from the tumour, its microenvironment and/or the host are detected by skeletal muscle at a very early stage and translated into alterations in mitochondrial energy generation. In patients with early‐stage CC, these effects were primarily characterized by a reduction in complex I activity, while, seemingly paradoxically, many RNA transcripts related to muscle oxidative respiration were increased. In patients with liver metastases, these mitochondrial changes were more pronounced at a functional level, with both complexes I and II activity being reduced. However, in this latter group, we did not observe significant transcriptional changes in muscle biopsies. A possible hypothesis is that reduced complex I activity in early‐stage CC activates an adaptive compensatory response, leading to upregulation of OxPhos‐related RNA transcripts. In metastatic patients, the exacerbation of complexes I and II reduction could then result in the loss of this adaptive response or, alternatively, could be a consequence of the lack of an adaptive response. Importantly, from a clinical perspective, the patients with primary‐stage CC and patients with colorectal cancer with liver metastases included in the study would be classified as non‐cachexic or pre‐cachexic [1], and the mitochondrial changes occurred before any noticeable alterations in physical function or inflammatory markers. Nevertheless, some of these mitochondrial changes correlated with physical performance indicators and biochemical parameters in the blood of the patients.

Due to the general population screening program for intestinal cancer that exists in the Netherlands, patients are often diagnosed at an early stage. This enabled us to include patients in our study who underwent surgery while they were still paucisymptomatic (presenting with an occult blood faecal test). This also applies to the potential effects on their muscle tissue, and it is possible that not all these patients would have developed cachexia if left untreated. However, understanding the early effects of cancer on OXPHOS could also help in gaining insights into energy metabolism and physical functioning‐related outcomes, such as the onset of fatigue [18, 19].

An important challenge of our observational trial in patients with cancer was to find appropriate controls [20]. In this study, we opted for an age‐matched control group consisting of patients without cancer. This brings our study in line with studies conducted in animal models, showing early changes related to cancer‐induced cachexia [5]. Such studies can make use of non‐diseased (sham) control animals [21, 22]. Previous human studies investigating early transcriptomic and functional mitochondrial and metabolic changes used weight‐stable patients with non‐cachectic cancer as controls [11, 12]. This aspect will also need to be considered in future studies when investigating the intricate molecular and physiological processes in patients with pre‐ and non‐cachectic cancer. One potential approach, though more taxing for the patient, could be to analyse multiple biopsies taken over a specific period.

The molecular mechanisms governing muscle wasting in cancer have predominantly been studied in animal models. These studies also report tumour‐induced effects on mitochondria in skeletal muscle [5]. In comparison, there is very limited data from human studies. Clinical studies exploring the role of mitochondrial function in skeletal muscle from patients with cancer at different stages are very scarce, and existing studies have primarily focused on modifications of mitochondrial biogenesis transcripts and protein expression [13, 14]. Most research primarily focuses on late‐stage cachectic cancers [23, 24]. Previous work in a cachectic rat model has shown that decreased complex IV activity can result in diminished muscle mitochondrial oxidative capacities [8]. In patients with cancer undergoing chemotherapy, similar mitochondrial function alterations have been observed in peripheral blood mononuclear cells (PBMC) [25], associated with OXPHOS complex III‐linked oxygen consumption defects [25]. However, caution is needed when determining whether these changes in OXPHOS complexes are induced by anti‐cancer treatments and whether they can be translated to muscle.

Our results may contribute to the development of new strategies for the early diagnosis of cancer‐induced muscle loss, which is crucial for preventing impaired physical functioning and improving treatment outcomes [26]. Current diagnostic criteria regarding cachexia primarily rely on anthropometric endpoints such as weight loss and muscle mass loss [7]. These criteria have limitations: 1) physical changes are often absent in early stages; 2) diagnosis is complicated by pre‐existing muscle loss or obesity [27], leading to delayed detection and missed opportunities for early intervention.

Our findings underscore these limitations, showing that despite all patients with CC being clinically categorized as non‐cachexia or pre‐cachexia according to the cancer cachexia criteria [1, 17, 28], mitochondrial dysfunction was already present in the *rectus abdominus* muscle. Interestingly, these patients exhibited physical characteristics similar to control patients. Elevated CRP levels, which are associated with cachexia development in various cancer types [29, 30], were only found to be increased in patients with colorectal cancer with liver metastases [29, 30]. At the same time, serum albumin, a negative acute phase protein, and considered another promising marker for cancer cachexia [31] remained unchanged in patients with liver metastases but was significantly lower in patients with primary CC. From this, we conclude that mitochondrial dysfunction precedes changes in markers such as CRP and albumin, particularly in patients with primary CC. Together with the observed correlations between mitochondrial function and physical and haematological parameters, our results underline the relevance of assessing mitochondrial dysfunction in cancer cachexia as a predictive indicator for future muscle mass and function loss in early‐stage CC.

Several potential markers have been proposed as possible indicators for earlier detection of cancer cachexia and sarcopenia, although their consistency varies across cancer types [32]. An intriguing question arises regarding whether a combined analysis of these marker levels alongside mitochondrial dysfunction could yield a more robust and reliable indicator for clinical applications. The answer to this might depend on the impact of the changes in mitochondrial function and the capability and time needed to recover from it. The patients with primary CC mainly showed muscle complex I dysfunction, while patients with liver metastases showed both complexes I and II dysfunctions. This difference might influence the muscle's recovery time and capability. In the present study, we compared two stages of CC. Future research should consider more specific population classifications based on cancer progression and cancer type. Additionally, a long‐term follow‐up of patients with primary CC could provide insights into differences in mitochondrial function recovery between those who achieve remission and those who develop (liver) metastases.

Our study lacks sex balance, particularly in the patients with primary CC group, due to a higher reluctance of women to participate in the study. In the RNAseq analysis of OXPHOS gene expression, females comprised 36% of the control group, 17% of the patients with primary CC group and 44% of the patients with liver metastatic group. The impact of sex on skeletal muscle mitochondrial respiration remains inconclusive. In mice, studies revealed higher mitochondrial mRNA expression in females [33]. Similarly, a human study identified higher transcript abundance of genes involved in mitochondrial OXPHOS and fatty acid oxidation in females [34]. In our previous human study, we observed qualitative differences in skeletal muscle aging between males and females, but no significant quantitative differences [35]. Despite fewer females in the patients with primary CC group, our results show significantly higher gene expression in the patients with primary CC group compared to controls, indicating upregulated gene expression in the patients with primary CC group. In the mitochondrial oxygen consumption test, females comprised 50% of the control group, 43% of the patients with primary CC group and 60% of the patients with liver metastatic group. Although the study is not powered to analyse sex differences, we found no evidence supporting such differences, consistent with a human study, where complex I‐, II‐, III‐ and IV‐dependent respiration in the gastrocnemius muscle showed no sex differences in muscle respiration among healthy individuals [36].

A limitation of this study is the unavoidable use of anaesthetics during muscle biopsy, which inhibits substrate oxidation in human muscle mitochondria without directly affecting the mitochondrial respiratory chain complexes or uncoupling oxidative phosphorylation [37]. To minimize variability, all patient cohorts underwent the same anaesthetic protocol. Biopsies of the *rectus abdominus* muscle were taken immediately after the first incision to further reduce the potential effects of anaesthetics. This human observational study was cross‐sectional in design. The originally planned one‐year follow‐up questionnaire could not be collected since COVID‐19 impacted the study during its initial phase.

Based on our data, we propose mitochondrial dysfunction could be used as a predictive marker for early‐stage diagnosis of cancer‐induced muscle dysfunction and muscle mass loss, potentially signalling the onset of cancer‐associated long‐term fatigue. Since our experimental measurements are labour‐intensive and not feasible on a large scale due to the need for immediate sample processing, it is essential to explore more practical biomarkers. Given the invasiveness of muscle biopsies, it would be valuable to determine whether mitochondrial function in PBMC correlates with muscle function. Near‐infrared spectroscopy (NIRS) could be applied. This non‐invasive portable method has been validated for mitochondrial function with OXPHOS gene expression changes in skeletal muscle [38]. Our findings also raise the question of whether complex I deficiency in mitochondria could be exploited for therapeutic purposes. Drugs targeting complex I in tumour cells exist and have been proposed for cancer treatment [39]. Future studies should investigate whether therapeutic strategies targeting mitochondrial dysfunction in skeletal muscle could help prevent cancer‐induced muscle wasting. Personalized interventions combining physical activity with pharmacological and nutritional support show promise [40]. However, a deeper understanding of the mechanisms underlying cancer‐related mitochondrial dysfunction is crucial for developing effective multimodal interventions.

## Author Contributions

K.V.N., R.P., S.G. and T.V.H. designed and optimized the study. X.L., M.V.D.E., H.M., R.P., M.P., S.L. and the COMUNEX group performed experiments. X.L. acquired the data present in this manuscript on physical characteristics, blood parameters, Oroboros respiration, and RNA sequencing. X.L., K.V.N. and S.G. analysed the data. X.L. wrote the manuscript with input from the other authors. S.G., K.V.N., R.F.W. and J.K. reviewed and edited the manuscript. All authors approved the final version of the manuscript.

## Conflicts of Interest

The authors declare no conflicts of interest.

## Supporting information


**Figure S1:** Related to Figure 4. Muscle OXPHOS transcripts exhibit no changes in patients with liver metastases. Heatmap displaying differentially expressed genes of (a) Complex I, (b) Complex II, (c) Complex III, (d) Cytochrome C (Cycs, Hccs) and Coenzyme Q metabolism, (e) Complex IV, (f) Complex V between patients with liver metastases and control patients. Genes were differentially regulated using a *p*‐value of 0.05. Control patients: *N* = 11 (male patients: *N* = 7, female patients: *N* = 4); patients with liver metastases: *N* = 9 (male patients: *N* = 5, female patients: *N* = 4). Colours reflect the level of gene expression.
**Figure S2:** Related to Figure 4. Heatmap displaying differentially expressed genes of (a) Complex I, (b) Complex II, (c) Complex III, (d) Cytochrome C (Cycs, Hccs) and Coenzyme Q metabolism, (e) Complex IV, (f) Complex V between patients with liver metastases and patients with primary CC. Genes were differentially regulated using an adjusted *p*‐value of 0.05 (adjusted according to Benjamini–Hochberg). Patients with primary colon cancer: *N* = 29 (male patients: *N* = 24, female patients: *N* = 5); patients with liver metastases: *N* = 9 (male patients: *N* = 5, female patients: *N* = 4). Colours reflect the level of gene expression.
**Figure S3:** Related to Figure 4. Quantitative PCR (qPCR) analysis of human muscle samples displaying relative mRNA expression levels of (a) Ndufa1 (representative gene of Complex I), (b) Ndufb1 (representative gene of Complex I), (c) Sdhd (representative gene of Complex II), (d) Uqcrh (representative gene of Complex III), (e) Cox5b (representative gene of Complex IV) and (f) Atp5me (representative gene of Complex V), normalized to the housekeeping gene 36B4. Control patients: *N* = 10 (male patients: *N* = 6, female patients: *N* = 4); patients with primary colon cancer: *N* = 28 (male patients: *N* = 23, female patients: *N* = 5); patients with liver metastases: *N* = 9 (male patients: *N* = 5, female patients: *N* = 4). All data were expressed as mean ± SEM and were analysed by one‐way ANOVA with Bonferroni's post hoc test. *p ≤ 0.05, **p ≤ 0.01 and ***p ≤ 0.001, ****p ≤ 0.0001.
**Figure S4:** Related to Figure 5. Representative respiration traces in the Complex I and Complex II‐linked OXPHOS state by Oroboros 2 k‐Oxygraph. Oxygen fluxes per chamber volume are depicted for the O2k chamber operated simultaneously: control patient = blue, patient with primary colon cancer = red, patient with liver metastases = green. Substrate and coupling state: tissue, Pyruvate + Malate (Py + Ma), ADP + Mg^2+^ (ADP), Cytochrome C (CytoC), Glutamate (Glu), Succinate (Suc), Carbonyl cyanide m‐chlorophenylhydrazone (CCCP), Rotenone, Antimycin A. Complex I and Complex II‐linked OXPHOS respiration stages are presented as LEAK‐N, OXPHOS‐N, OXPHOS‐NS, ET‐NS and ET‐S.
**Figure S5:** Related to Figure 5. Mitochondrial oxygen consumption in (a) male patients and (b) female patients presented with respiration stages: LEAK‐N, OXPHOS‐N, OXPHOS‐NS, ET‐NS and ET‐S. Muscle tissues of control patients: *N* = 4 (male patients: *N* = 2, female patients: *N* = 2); patients with primary colon cancer: *N* = 7 (male patients: *N* = 4, female patients: *N* = 3); Patients with liver metastases: *N* = 5 (male patients: *N* = 2, female patients: *N* = 3). All data were expressed as mean ± SEM. Data were analysed by two‐way ANOVA with Tukey post hoc test. *p ≤ 0.05, **p ≤ 0.01 and ***p ≤ 0.001, ****p ≤ 0.0001.
**Figure S6:** Related to Table 4. Pearson correlation coefficient heat map of mutual analysis between 36 parameters of all patients. The values in the square lattices represent the magnitude of R value of correlation analysis displayed by colour difference. Control patients: *N* = 17 (male patients: *N* = 11, female patients: *N* = 6); patients with primary colon cancer: *N* = 30 (male patients: *N* = 25, female patients: *N* = 5); patients with liver metastases: *N* = 10 (male patients: *N* = 5, female patients: *N* = 5).
**Figure S7:** Related to Table 4. Pearson correlation coefficient heat map of mutual analysis between mitochondrial function and other parameters (r above 0.5 are shown). The values in the square lattices represent the magnitude of the R value of correlation analysis displayed by colour difference meanwhile. Control patients: *N* = 17 (male patients: *N* = 11, female patients: *N* = 6); patients with primary colon cancer: *N* = 30 (male patients: *N* = 25, female patients: *N* = 5); patients with liver metastases: *N* = 10 (male patients: *N* = 5, female patients: *N* = 5).

**Data S1:** Supplementary Information.
