# Supplementary material for: Primary‐Stage Colon Cancer Impairs Muscle Energy Metabolism by Suppressing Mitochondrial Complex I Activity
Source: J Cachexia Sarcopenia Muscle. 2025 Nov 12;16(6):e70117. doi: 10.1002/jcsm.70117 (PMC12605958; doi:10.1002/jcsm.70117)
Supplement: Supplementary file 1 — Figure S1: Related to Figure 4. Muscle OXPHOS transcripts exhibit no changes in patients with liver metastases. Heatmap displaying differentially expressed genes of (a) Complex I, (b) Complex II, (c) Complex III, (d) Cytochrome C (Cycs, Hccs) and Coenzyme Q metabolism, (e) Complex IV, (f) Complex V between patients with liver metastases and control patients. Genes were differentially regulated using a p‐value of 0.05. Control patients: N = 11 (male patients: N = 7, female patients: N = 4); patients with liver metastases: N = 9 (male patients: N = 5, female patients: N = 4). Colours reflect the level of gene expression. Figure S2: Related to Figure 4. Heatmap displaying differentially expressed genes of (a) Complex I, (b) Complex II, (c) Complex III, (d) Cytochrome C (Cycs, Hccs) and Coenzyme Q metabolism, (e) Complex IV, (f) Complex V between patients with liver metastases and patients with primary CC. Genes were differentially regulated using an adjusted p‐value of 0.05 (adjusted according to Benjamini–Hochberg). Patients with primary colon cancer: N = 29 (male patients: N = 24, female patients: N = 5); patients with liver metastases: N = 9 (male patients: N = 5, female patients: N = 4). Colours reflect the level of gene expression. Figure S3: Related to Figure 4. Quantitative PCR (qPCR) analysis of human muscle samples displaying relative mRNA expression levels of (a) Ndufa1 (representative gene of Complex I), (b) Ndufb1 (representative gene of Complex I), (c) Sdhd (representative gene of Complex II), (d) Uqcrh (representative gene of Complex III), (e) Cox5b (representative gene of Complex IV) and (f) Atp5me (representative gene of Complex V), normalized to the housekeeping gene 36B4. Control patients: N = 10 (male patients: N = 6, female patients: N = 4); patients with primary colon cancer: N = 28 (male patients: N = 23, female patients: N = 5); patients with liver metastases: N = 9 (male patients: N = 5, female patients: N = 4). All data were expressed as mean ± [file JCSM-16-e70117-s002.docx]

# Primary-stage colon cancer impairs muscle energy metabolism by suppressing mitochondrial complex I activity

# Xiaolin Li^1^; Miranda van der Ende^1,2^; Hanneke Moonen^1,3^; Rogier Plas^1^; Susanne Lotstra^1^; Mieke Poland^1^; the COMUNEX group^†^; Jaap Keijer^2^; Renger F. Witkamp^1^; Tjarda van Heek^1,3^; Sander Grefte^2^; and Klaske van Norren^1,*^

1. Division of Human Nutrition and Health, Wageningen University, Wageningen, The Netherlands
2. Human and Animal Physiology, Wageningen University, Wageningen, The Netherlands
3. Gelderse Vallei Hospital, Ede, The Netherlands

† The COMUNEX group consists of the following authors: Flip M. Kruyt^3^, Colin Sietses^3^, Gabie M de Jong^3^, Roland MHG Mollen^3^, Joé LP Kolkert^3^, Dik Snijdelaar^3^, Marlieke Visser^3^, Jaap Dronkers^3^

* Correspondence: Klaske van Norren, Nutritional Biology, Division of Human Nutrition and Health, Wageningen University, Wageningen, The Netherlands. Email: [klaske.vannorren@wur.nl](mailto:klaske.vannorren@wur.nl).

# Supplemental Material

## Supplementary Figure 1. Related to Figure 4.


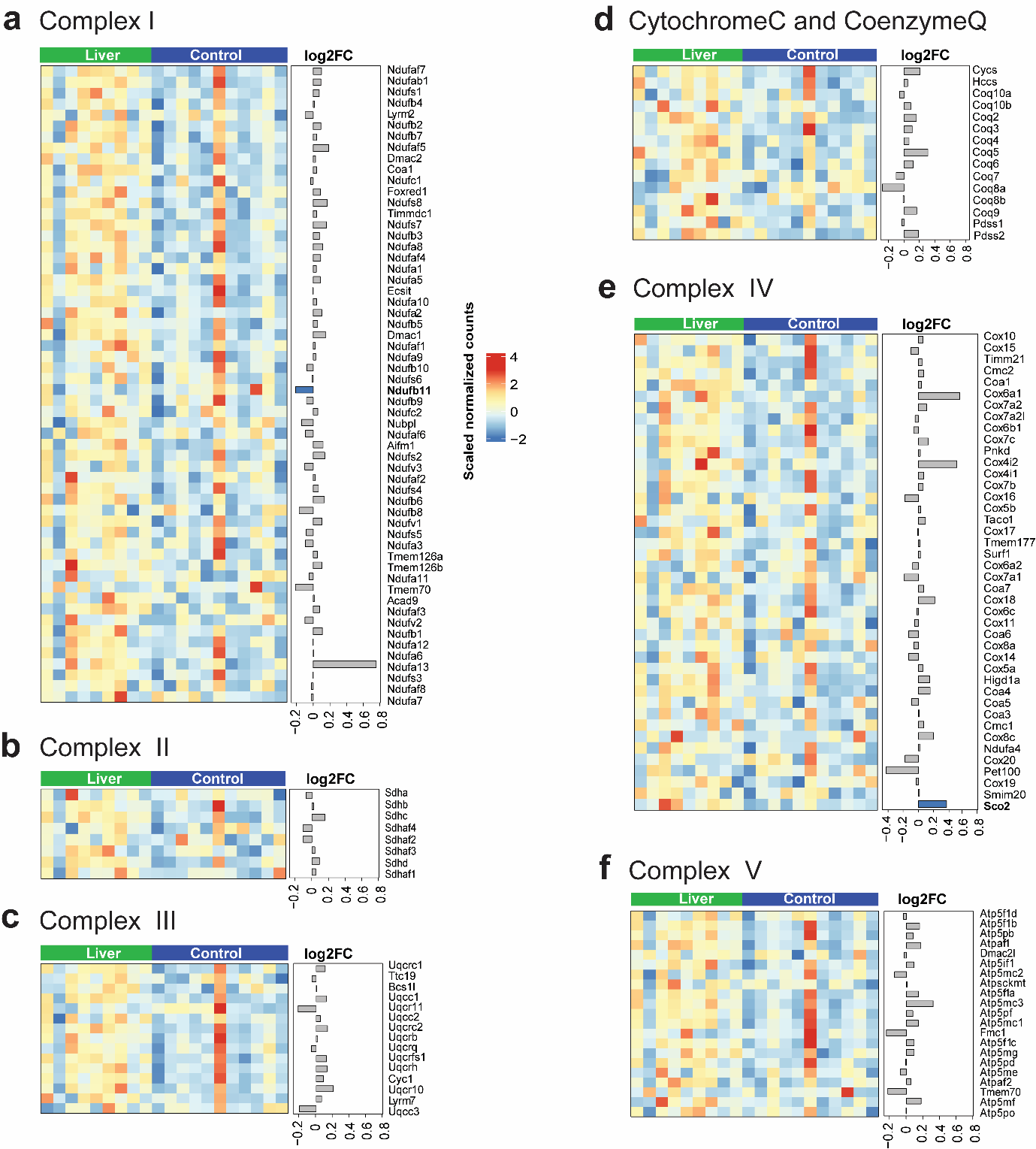


**Supplementary Figure 1. Related to Figure 4. Muscle OXPHOS transcripts exhibit no changes in liver metastases patients.** Heatmap displaying differentially expressed genes of **a** Complex I, **b** Complex II, **c** Complex III, **d** Cytochrome C (Cycs, Hccs) and Coenzyme Q metabolism, **e** Complex IV, **f** Complex V between patients with liver metastases and control patients. Genes were differentially regulated using a P-value of 0.05. Control patients: N=11 (male patients: N=7, female patients: N=4); Patients with liver metastases: N=9 (male patients: N=5, female patients: N=4). Colors reflect the level of gene expression.

## Supplementary Figure 2. Related to Figure 4.


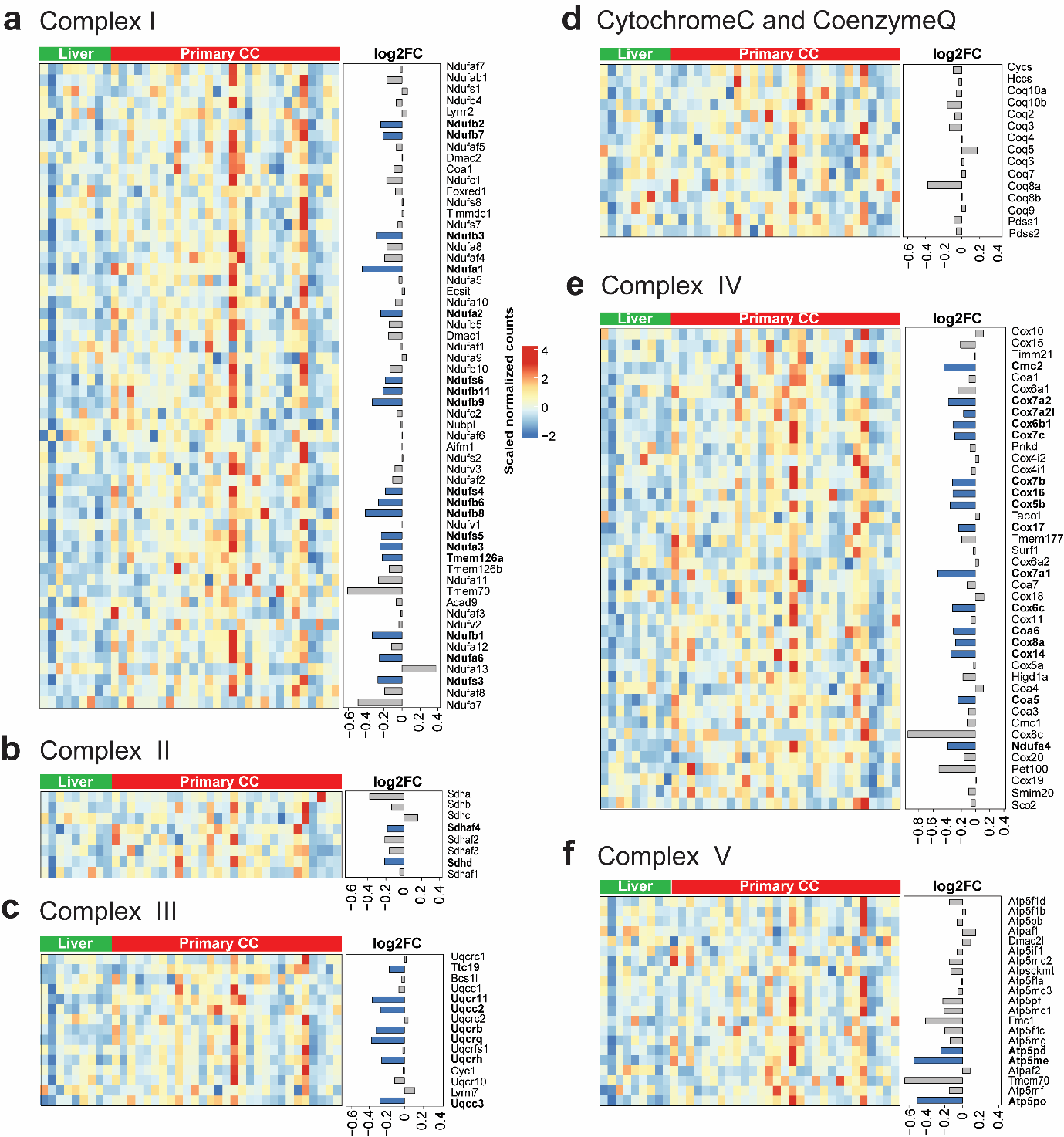


**Supplementary Figure 2. Related to Figure 4.** Heatmap displaying differentially expressed genes of **a** Complex I, **b** Complex II, **c** Complex III, **d** Cytochrome C (Cycs, Hccs) and Coenzyme Q metabolism, **e** Complex IV, **f** Complex V between patients with liver metastases and primary CC patients. Genes were differentially regulated using an adjusted P-value of 0.05 (adjusted according to Benjamini–Hochberg). Primary colon cancer patients: N=29 (male patients: N=24, female patients: N=5); Patients with liver metastases: N=9 (male patients: N=5, female patients: N=4). Colors reflect the level of gene expression.

## Supplementary Figure 3. Related to Figure 4.


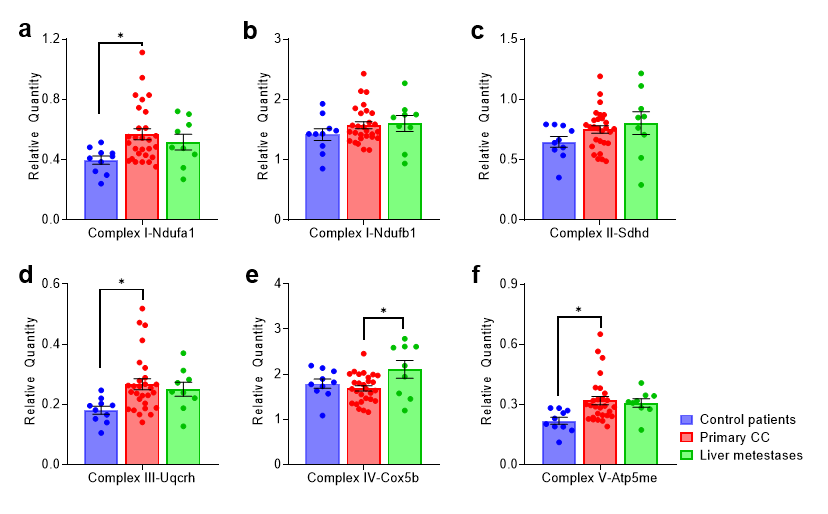


**Supplementary Figure 3. Related to Figure 4.** Quantitative PCR (qPCR) analysis of human muscle samples displaying relative mRNA expression levels of **a** Ndufa1 (representative gene of Complex I), **b** Ndufb1 (representative gene of Complex I), **c** Sdhd (representative gene of Complex II), **d** Uqcrh (representative gene of Complex III), **e** Cox5b (representative gene of Complex IV), and **f** Atp5me (representative gene of Complex V), normalized to the housekeeping gene 36B4. Control patients: N=10 (male patients: N=6, female patients: N=4); Primary colon cancer patients: N=28 (male patients: N=23, female patients: N=5); Patients with liver metastases: N=9 (male patients: N=5, female patients: N=4). All data were expressed as mean ± SEM and were analyzed by one-way ANOVA with Bonferroni‘s post hoc test. **p* ≤ 0.05, ***p* ≤ 0.01, and ****p*≤ 0.001, *****p*≤ 0.0001.

## Supplementary Figure 4. Related to Figure 5.


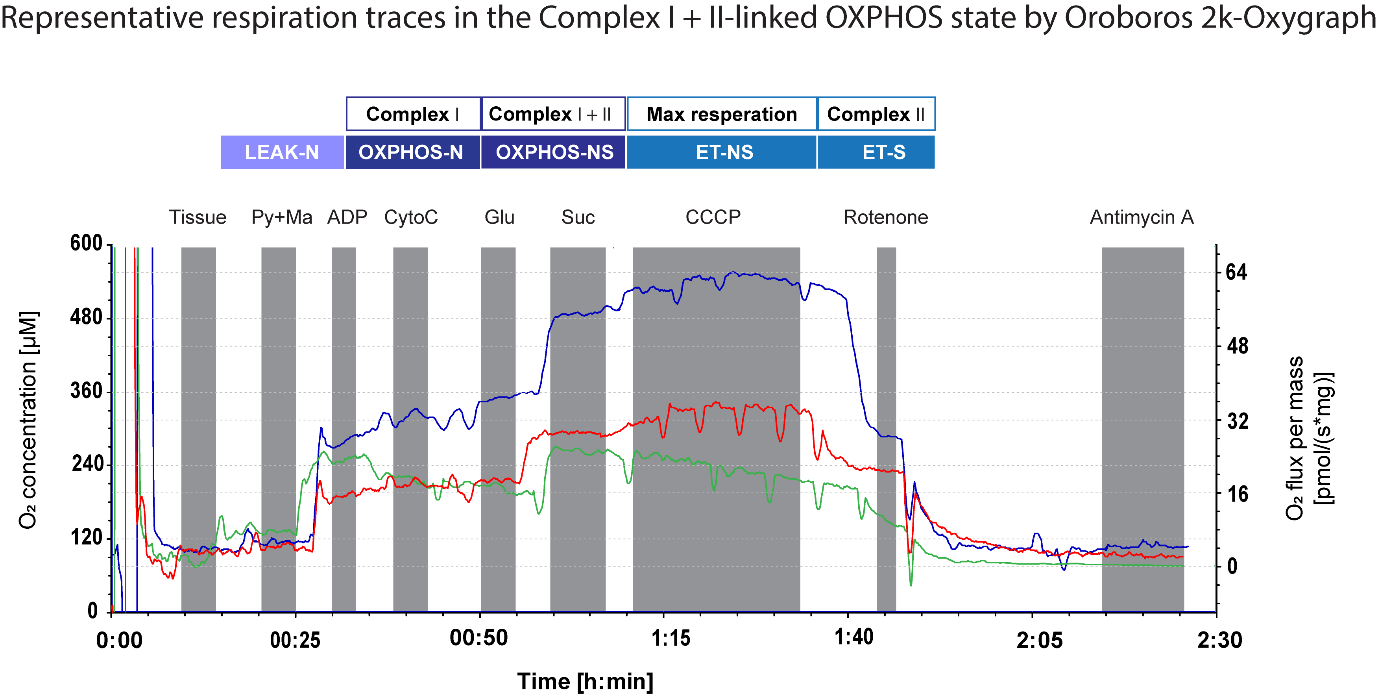


**Supplementary Figure 4. Related to Figure 5. Representative respiration traces in the Complex I and Complex II-linked OXPHOS state by Oroboros 2k-Oxygraph.** Oxygen fluxes per chamber volume are depicted for the O2k chamber operated simultaneously: control patient=blue, primary colon cancer patient=red, patient with liver metastases=green. Substrate and coupling state: tissue, Pyruvate+Malate (Py+Ma), ADP+Mg^2+^ (ADP), Cytochrome C (CytoC), Glutamate (Glu), Succinate (Suc), Carbonyl cyanide m-chlorophenylhydrazone (CCCP), Rotenone, Antimycin A. Complex I and Complex II-linked OXPHOS respiration stages are presented as LEAK-N, OXPHOS-N, OXPHOS-NS, ET-NS, and ET-S.

## Supplementary Figure 5. Related to Figure 5.


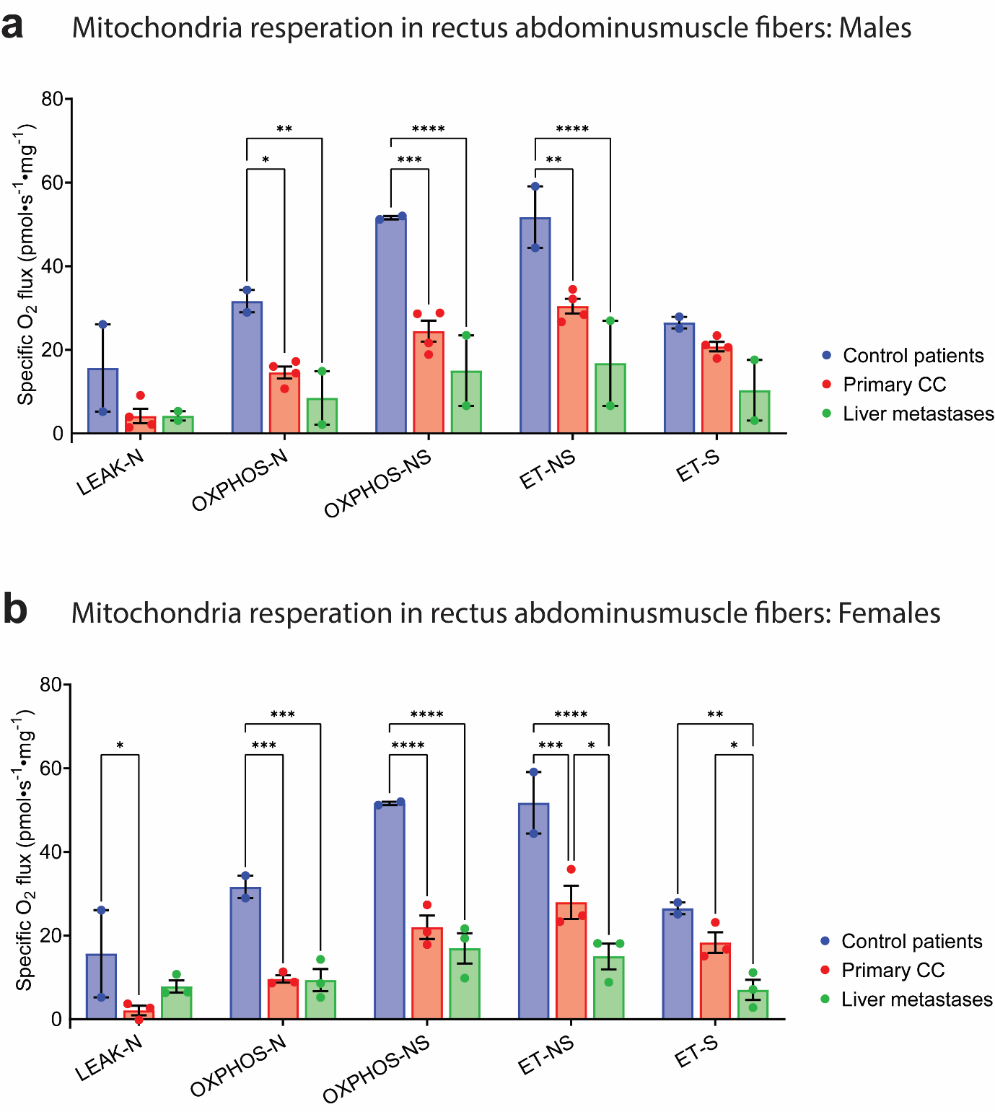


**Supplementary Figure 5. Related to Figure 5.** Mitochondrial oxygen consumption in **a** male patients and **b** female patients presented with respiration stages: LEAK-N, OXPHOS-N, OXPHOS-NS, ET-NS, and ET-S. Muscle tissues of control patients: N=4 (male patients: N=2, female patients: N=2); Primary colon cancer patients: N=7 (male patients: N=4, female patients: N=3); Patients with liver metastases: N=5 (male patients: N=2, female patients: N=3). All data were expressed as mean ± SEM. Data were analyzed by two-way ANOVA with Tukey post hoc test. **p*≤0.05, ***p*≤0.01, and ****p*≤0.001, *****p*≤0.0001.

## Supplementary Figure 6. Related to Table 3.


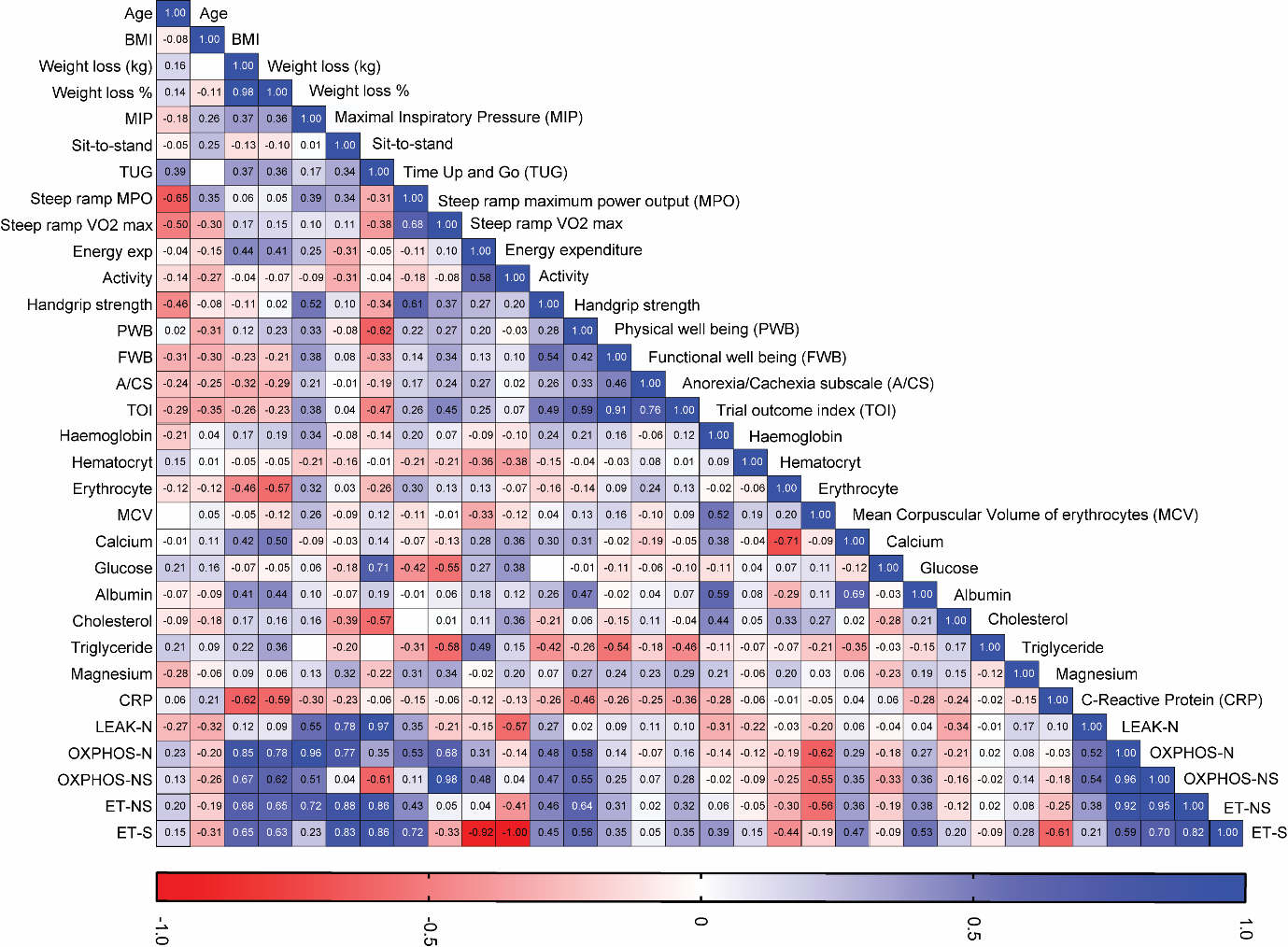
 **Supplementary Figure 6. Related to Table 3. Pearson correlation coefficient heat map of mutual analysis between 36 parameters of all patients.** The values in the square lattices represent the magnitude of R value of correlation analysis displayed by color difference. Control patients: N=17 (male patients: N=11, female patients: N=6); Primary colon cancer patients: N=30 (male patients: N=25, female patients: N=5); Patients with liver metastases: N=10 (male patients: N=5, female patients: N=5).

## Supplementary Figure 7. Related to Table 3.
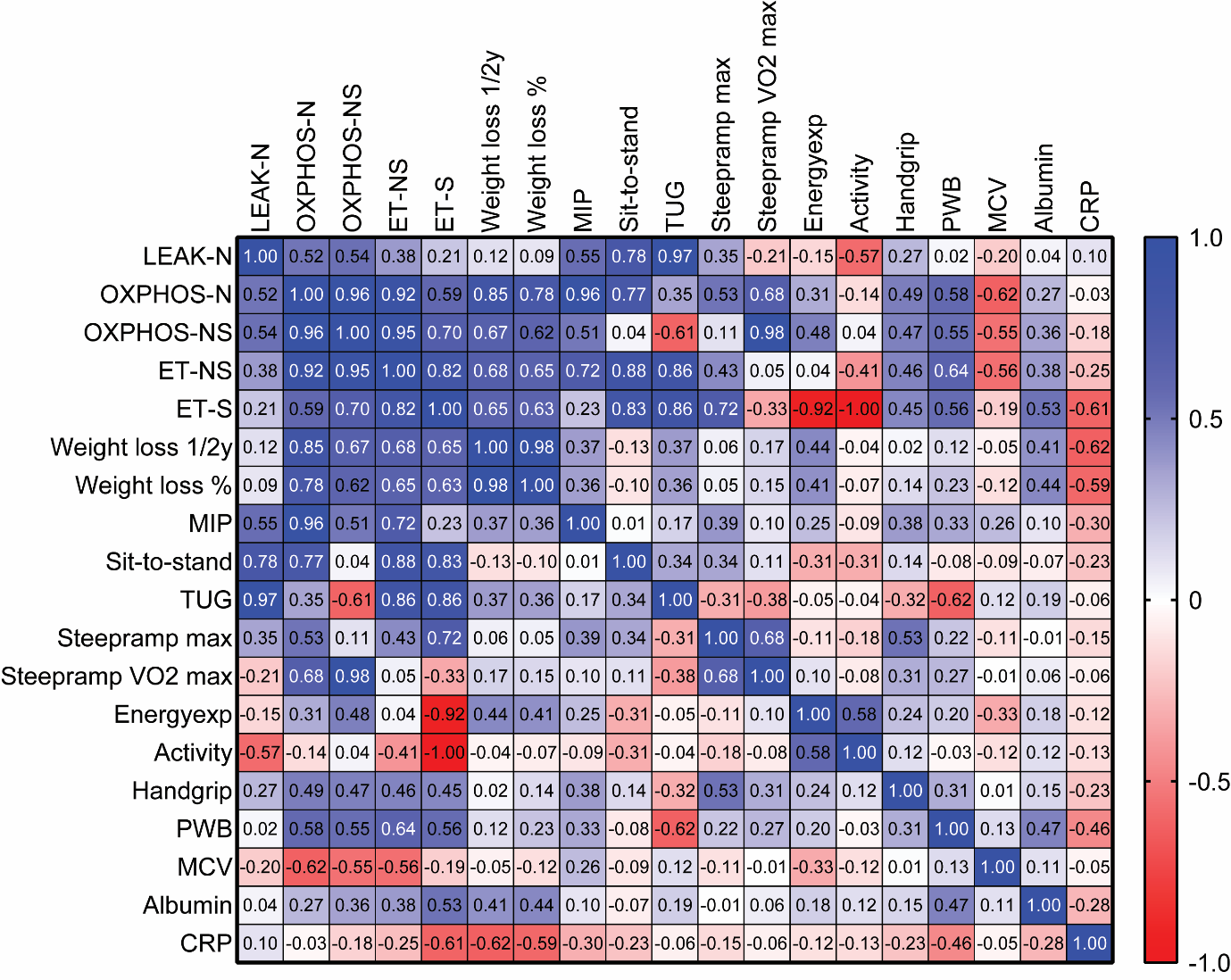


**Supplementary Figure 7. Related to Table 3. Pearson correlation coefficient heat map of mutual analysis between mitochondrial function and other parameters (r above 0.5 are shown).** The values in the square lattices represent the magnitude of the R value of correlation analysis displayed by color difference meanwhile. Control patients: N=17 (male patients: N=11, female patients: N=6); Primary colon cancer patients: N=30 (male patients: N=25, female patients: N=5); Patients with liver metastases: N=10 (male patients: N=5, female patients: N=5).
