# Supplementary material for: Primary‐Stage Colon Cancer Impairs Muscle Energy Metabolism by Suppressing Mitochondrial Complex I Activity
Source: J Cachexia Sarcopenia Muscle. 2025 Nov 12;16(6):e70117. doi: 10.1002/jcsm.70117 (PMC12605958; doi:10.1002/jcsm.70117)
Supplement: Supplementary file 2 — Data S1: Supplementary Information. [file JCSM-16-e70117-s001.docx]

# Primary-stage colon cancer impairs muscle energy metabolism by suppressing mitochondrial complex I activity

# Xiaolin Li^1^; Miranda van der Ende^1,2^; Hanneke Moonen^1,3^; Rogier Plas^1^; Susanne Lotstra^1^; Mieke Poland^1^; the COMUNEX group^†^; Jaap Keijer^2^; Renger F. Witkamp^1^; Tjarda van Heek^1,3^; Sander Grefte^2^; and Klaske van Norren^1,*^

1. Division of Human Nutrition and Health, Wageningen University, Wageningen, The Netherlands
2. Human and Animal Physiology, Wageningen University, Wageningen, The Netherlands
3. Gelderse Vallei Hospital, Ede, The Netherlands

† The COMUNEX group consists of the following authors: Flip M. Kruyt^3^, Colin Sietses^3^, Gabie M de Jong^3^, Roland MHG Mollen^3^, Joé LP Kolkert^3^, Dik Snijdelaar^3^, Marlieke Visser^3^, Jaap Dronkers^3^

* Correspondence: Klaske van Norren, Nutritional Biology, Division of Human Nutrition and Health, Wageningen University, Wageningen, The Netherlands. Email: [klaske.vannorren@wur.nl](mailto:klaske.vannorren@wur.nl).

# Methods

## 1. Compliance with ethical standards

Data was collected between May 2017 and March 2024 in two research centers in the Netherlands: Gelderse Vallei Hospital (Ede) and Wageningen University (Wageningen).

## 2. Study population

At the start of this study there was no standardized method to determine group size for gene expression of complete pathways as primary parameter. The sample size of this study was based on the estimated accuracy of gene expression comparisons derived from the literature. A previous study reported that a sample size of n=15 per group yielded a high prediction accuracy (0.8–0.9) for detecting sex differences in muscle gene expression associated with metabolic changes ^1^. Given that this study investigated the presence of a tumor versus no tumor, larger differences between groups were expected. Therefore, we estimated group size to be at least n=10 per group. A total of 30 primary colon cancer patients were included to enable analyses not only at the group level but also within subgroups. Additionally, ethical approval allowed for patient replacement if muscle samples could not be obtained. By including both primary colon cancer patients and those with liver metastases, the study aimed to represent a broad spectrum of muscle wasting and capture the heterogeneity in disease-related changes in muscle function and metabolism. To mitigate variability and address potential challenges in recruiting colon cancer patients, the control group was designed to reflect the patient distribution by sex. However, during recruitment, a higher number of male participants volunteered for the CC group. Consequently, the number of male controls was increased to 11, including one individual for whom a muscle biopsy was not possible.

## 3. Physical data and biochemical blood markers collection

Preoperative assessments included a validated Functional Assessment of Anorexia/Cachexia Therapy (FAACT) questionnaire, a physical function test, and a muscle function test. The FAACT questionnaire assessed physical and functional well-being and anorexia/cachexia using three subscales, contributing to the FAACT Trial Outcome Index (TOI). This questionnaire is validated for the specific target group ^2,3^. Physical function was evaluated on Day -1 or Day 0 using the Timed Up and Go (TUG) test, where patients were timed as they rose from an armchair, walked 3 meters, turned, returned, and sat down ^4^. Muscle function was measured on Day 0 by recording three consecutive hand grip strength readings for each hand using a Jamar® dynamometer, accurate to the nearest kilogram.

On the day of the surgery (Day 0) patients came in fasted for regular care procedures. Fasted blood samples (total 55 mL) were collected after inserting an intravenous cannula in the holding area (regular care) to identify levels of Hb, CRP, albumin, glucose, and other hematological parameters.

## 4. Muscle transcriptome analysis

### 4.1 Sample collection and RNA extraction

Muscle tissues collected for the transcriptome analysis included 11 control patients (male patients: N=7, female patients: N=4), 29 primary CC patients (male patients: N=24, female patients: N=5), and 9 patients with liver metastases (male patients: N=5, female patients: N=4). During the surgery, a biopsy (1 cm^3^) of the *rectus abdominus* muscle was taken. Biopsies were taken directly after the first incision of the surgery to minimize the possible effect of anesthetics. Most of the primary colon tumor resections were done using laparoscopic surgery. In the case of open surgery (most of the liver resections and surgery of controls), we took the muscle biopsy at once. Collected tissue was snap-frozen in liquid nitrogen, and stored at −80°C until further analysis.

Approximately 15 mg of frozen muscle tissue was added to a metal bead with 1 ml of Trizol and homogenized using a TissueLyser LT (Qiagen) at 50 Hz for 4 minutes to ensure complete lysis. The mixture was incubated by shaking at 1400 rpm at room temperature for 30 minutes until fully dissolved. The metal bead was removed, and the homogenate was centrifuged at 12,000 g for 10 minutes at 4°C. The supernatant was transferred to a clean 1.5 ml microcentrifuge tube. Cold chloroform (200 μl) was added, shaken thoroughly for 30 seconds, incubated on ice for 10 minutes, and centrifuged at 12,000 rpm for 15 minutes at 4°C (Mikro 200R, Hettich, Germany). The aqueous layer was transferred to a new tube, and 500 μl of ice-cold isopropanol was added, shaken thoroughly for 30 seconds, incubated on ice for 10 minutes, and centrifuged at 12,000 rpm for 30 minutes at 4°C. The supernatant was discarded. The pellet was washed three times with 1 ml of ice-cold 75% ethanol, inverted once, and rolled five times. Each wash was followed by centrifugation at 12,000 g for 15 minutes at 4°C and supernatant removal. The pellet was air-dried and dissolved in 40 μl of RNase/DNase-free water. The tube was immediately placed on ice. RNA concentration was measured using a NanoDrop spectrophotometer (Thermo Fisher). RNA integrity was assessed using RNA ScreenTape on the 2200 TapeStation (Agilent), with all samples having an RNA integrity number (RIN)>7.0 and a 260/280 ratio>1.8.

### 4.2 RNA sequencing and differential gene expression analysis

RNA preparation, library construction, and sequencing on Illumina NovaSeq 6000 were performed at Novogene (UK). Clean reads were obtained in FASTQ format, and quality check was performed using FASTQC ^5^. Reads were aligned to the human genome (GRch38.p13) using STAR ^6^, and counts were quantified using HTSeq ^7^. The average sequencing depth was 23 M paired-end reads, of which at least 92% were uniquely mapped.

Data analysis and statistical testing were performed in R version 4.3.3 and using appropriate Bioconductor packages. Differentially expressed genes (DEGs) between the groups were identified using DESeq2 ^8^. Genes with less than 10 reads per row were removed, and Benjamini–Hochberg multiple testing correction was used to obtain adjusted P-values; a false discovery rate (FDR) of 5% was accepted. Principal component analysis was done using variance stabilizing transformed (VST) data. Associations between gene expression and the continuous variable MVPA were done using the Limma-Voom ^9^. Normalization factors were calculated using calcNormfactors using EdgeR ^10^, and low-expressed genes were filtered. Voom was used to calculate the mean-variance relationship of the log counts, and linear model was fitted using weighted least squares for each gene. Empirical Bayes was used for smoothing of standard errors ^11^. Gene set enrichment analysis was done using clusterProfiler ^12^ for the “biological process” gene ontology gene sets. Gene sets were considered enriched with a Benjamini–Hochberg adjusted P-value < 0.05, and GO terms were filtered based on 75% overlap in genes. The human MitoCarta3.0 gene set was used as a reference inventory for mitochondrial genes ^13^.

# Reference

1. Stretch C, Khan S, Asgarian N, Eisner R, Vaisipour S, Damaraju S *et al.* Effects of sample size on differential gene expression, rank order and prediction accuracy of a gene signature. *PLoS One* 2013;**8**.

2. Ribaudo JM, Cella D, Hahn EA, Lloyd SR, Tchekmedyian NS, Von Roenn J *et al.* Re-validation and Shortening of the Functional Assessmentof Anorexia/Cachexia Therapy (FAACT) Questionnaire. *Quality of Life Research 2000 9:10* 2000;**9**:1137–1146.

3. Blauwhoff-Buskermolen S, Ruijgrok C, Ostelo RW, de Vet HCW, Verheul HMW, de van der Schueren MAE *et al.* The assessment of anorexia in patients with cancer: cut-off values for the FAACT–A/CS and the VAS for appetite. *Supportive Care in Cancer* 2016;**24**:661–666.

4. Podsiadlo D, Richardson S. The Timed “Up & Go”: A Test of Basic Functional Mobility for Frail Elderly Persons. *J Am Geriatr Soc* 1991;**39**:142–148.

5. Babraham Bioinformatics - FastQC A Quality Control tool for High Throughput Sequence Data. https://www.bioinformatics.babraham.ac.uk/projects/fastqc/. Accessed 22 May 2024.

6. Dobin A, Davis CA, Schlesinger F, Drenkow J, Zaleski C, Jha S *et al.* STAR: ultrafast universal RNA-seq aligner. *Bioinformatics* 2013;**29**:15–21.

7. Anders S, Pyl PT, Huber W. HTSeq—a Python framework to work with high-throughput sequencing data. *Bioinformatics* 2015;**31**:166–169.

8. Love MI, Huber W, Anders S. Moderated estimation of fold change and dispersion for RNA-seq data with DESeq2. *Genome Biol* 2014;**15**:1–21.

9. Ritchie ME, Phipson B, Wu D, Hu Y, Law CW, Shi W *et al.* limma powers differential expression analyses for RNA-sequencing and microarray studies. *Nucleic Acids Res* 2015;**43**:e47–e47.

10. Robinson MD, McCarthy DJ, Smyth GK. edgeR: a Bioconductor package for differential expression analysis of digital gene expression data. *Bioinformatics* 2010;**26**:139–140.

11. Smyth GK. Linear models and empirical bayes methods for assessing differential expression in microarray experiments. *Stat Appl Genet Mol Biol* 2004;**3**.

12. Yu G, Wang LG, Han Y, He QY. ClusterProfiler: An R package for comparing biological themes among gene clusters. *OMICS* 2012;**16**:284–287.

13. Rath S, Sharma R, Gupta R, Ast T, Chan C, Durham TJ *et al.* MitoCarta3.0: an updated mitochondrial proteome now with sub-organelle localization and pathway annotations. *Nucleic Acids Res* 2021;**49**:D1541–D1547.

14. Pesta D, Gnaiger E. High-resolution respirometry: OXPHOS protocols for human cells and permeabilized fibers from small biopsies of human muscle. *Methods in Molecular Biology* 2012;**810**:25–58.
